# Genome Anatomy of *Pyrenochaeta unguis-hominis* UM 256, a Multidrug Resistant Strain Isolated from Skin Scraping

**DOI:** 10.1371/journal.pone.0162095

**Published:** 2016-09-14

**Authors:** Yue Fen Toh, Su Mei Yew, Chai Ling Chan, Shiang Ling Na, Kok Wei Lee, Chee-Choong Hoh, Wai-Yan Yee, Kee Peng Ng, Chee Sian Kuan

**Affiliations:** 1 Department of Medical Microbiology, Faculty of Medicine, University of Malaya, Kuala Lumpur, Malaysia; 2 Codon Genomics SB, Seri Kembangan, Selangor Darul Ehsan, Malaysia; Louisiana State University, UNITED STATES

## Abstract

*Pyrenochaeta unguis-hominis* is a rare human pathogen that causes infection in human skin and nail. *P*. *unguis-hominis* has received little attention, and thus, the basic biology and pathogenicity of this fungus is not fully understood. In this study, we performed in-depth analysis of the *P*. *unguis-hominis* UM 256 genome that was isolated from the skin scraping of a dermatitis patient. The isolate was identified to species level using a comprehensive multilocus phylogenetic analysis of the genus *Pyrenochaeta*. The assembled UM 256 genome has a size of 35.5 Mb and encodes 12,545 putative genes, and 0.34% of the assembled genome is predicted transposable elements. Its genomic features propose that the fungus is a heterothallic fungus that encodes a wide array of plant cell wall degrading enzymes, peptidases, and secondary metabolite biosynthetic enzymes. Antifungal drug resistance genes including *MDR*, *CDR*, and *ERG11/CYP51* were identified in *P*. *unguis-hominis* UM 256, which may confer resistance to this fungus. The genome analysis of *P*. *unguis-hominis* provides an insight into molecular and genetic basis of the fungal lifestyles, understanding the unrevealed biology of antifungal resistance in this fungus.

## Introduction

*Pyrenochaeta* is a genus of dematiaceous coelomycetes belonging to the order of Pleosporales in the class of Dothideomycetes [1]. Based on the recent taxonomic revision by de Gruyter *et al*. [1], *Pyrenochaeta romeroi* and *Pyrenochaeta mackinnonii* were excluded from the *Pyrenochaeta* genus [1]. To date, nearly 20 *Pyrenochaeta* species are recognized. The members of the genus *Pyrenochaeta* are ubiquitously found in the environment as saprophyte in soil, plant and wood, particularly in tropical and subtropical area. Several are causes of devastating plant diseases, including corky root rot disease in tomato, corn and maize, leading to significant yield loss in the crops worldwide [2–5].*Pyrenochaeta* species are rarely involved in human infection. The human pathogenic *Pyrenochaeta* species include *Pyrenochaeta keratinophilia* [6] and *Pyrenochaeta unguis-hominis* [7, 8] that have been isolated from the skin, nails and corneal scarping.

*P*. *unguis-hominis* was first isolated from toe-nails of a male patient in 1975 [8]. It is a rare human pathogen that has been reported so far in only two cases of nail infection [7, 8]. Over the last five years, the University of Malaya Medical Centre (UMMC), a tertiary hospital in Kuala Lumpur, the capital of Malaysia, reported only one *P*. *unguis-hominis* isolated from the superficial skin [9]. At the initial stage, the identification of the clinical isolate to the species level is hampered by ITS sequencing due to lack of ITS reference sequences in the public databases. In this study, a combined detailed morphological examination and multilocus phylogeny analysis enabled us to confirm the isolate as *P*. *unguis-hominis*. The *in vitro* drug susceptibility test revealed that this fungus was resistance to multiple antifungal agents, including echinocandin and azoles [9]. Thus, *P*. *unguis-hominis* UM 256 can be a fungal model to elucidate molecular mechanisms of drug resistance in pathogenic fungi.

*P*. *unguis-hominis* has received little attention and thus, its extra-human habitat and the underlying pathogenicity are unknown. To provide a better understanding of its basic biology, a draft genome sequence of the multidrug resistant strain was generated [10]. In this work, we report a comprehensive analysis of this genome and its gene content. Thus we generate knowledge about biology, lifestyle, and fundamental pathogenicity mechanism of *P*. *unguis-hominis*.

## Materials and Methods

### Ethics statement

The genome used in this study was obtained from a fungal isolate which was isolated in the year 2010. The isolate was routinely cultured and archived by the mycology laboratory in University Malaya Medical Centre [9]. The authors were not involved in the work dealing with human or animal subjects, thus ethical clearance is exempted for this study.

### Fungal isolate

*P*. *unguis-hominis* UM 256 was isolated from the skin scraping of a dermatitis patient. The fungal isolate was grown on Sabouraud Dextrose agar (SDA) at 30°C for up to 14 days. Macroscopic and microscopic examination of the isolate were carried out as previously described [9].

### Multilocus phylogenetic analysis

The internal transcribed spacer (ITS) [11], large ribosomal subunit (LSU) [12, 13], and small ribosomal subunit (SSU) [11] were used as the targets in UM 256 molecular identification. The DNA extraction and multilocus phylogenetic analysis were performed as described by Kuan *et al*. [14]. The phylogenetic tree was constructed using a total of 14 fungal sequences including 12 *Pyrenochaeta* spp. sequences obtained from NCBI GenBank and two strains (*Chaetosphaeronema hispidulum* and *Setophoma terrestris*) were used as outgroups (Table 1). Multiple sequences alignments of all collected ITS, LSU and SSU sequences were generated individually using M-Coffee [15] and then joined together for Bayesian Markov Chain Carlo (MCMC) analysis partitioned by gene. Bayesian tree analyses were performed using MrBayes v3.2.1 [16] using reversible jump MCMC averaging over the general time reversible (GTR) and gamma-distributed rate for all partitioned scheme subsets, fixing the frequencies of the stationary state to be equal. A total of 1,000,000 generations were run with a sampling frequency of 100, and diagnostics were total up with every 1,000 generations. The first 250 trees were then discarded by a 25% burn-in setting. Convergence was assessed with the standard deviation of split frequencies that below 0.01, trend for the generation plot versus the log probability of the data were not obvious, and the potential scale reduction factor (PSRF) close to 1.0 for all parameters.

**Table 1:** List of *Pyrenochaeta* spp. fungus sequences (ITS, SSU, and LSU) obtained from NCBI and Q-bank for phylogenetic trees.

Species name	Strain number	Genbank accession		
		ITS	SSU	LSU
*Pyrenocheata acicula*	CBS 101634	*	GQ387542	GQ387603
	CBS 124142	*	GQ387543	GQ387604
	CBS 812.95	*	GQ387541	GQ387602
*Pyrenochaeta berberidis*	CBS 363.93	JF740191	GQ387545	GQ387606
	CBS 394.84	*	GQ387544	GQ387605
*Pyrenochaeta cava*	CBS 115979	AY853248	EU754099	EU754198
	CBS 257.68	JF740260	EU754100	EU754199
*Pyrenochaeta corni*	CBS 234.92	-	EU754077	EU754176
	CBS 248.79	-	GQ387547	GQ387608
*Pyrenochaeta gentinicola*	MAFF 425531	AB499790	-	-
*Pyrenochaeta keratinophila*	CBS 123295	EU885415	-	-
*Pyrenochaeta lycopersici*	CBS 306.65	-	EU754106	GQ387612
	CBS 267.59	JF740261	GQ387551	EU754205
*Pyrenochaeta nobilis*	CBS 407.76	EU930011	EU754107	EU754206
	CBS 566.75	*	GQ387555	GQ387616
*Pyrenochaeta inflorescentiae*	CBS 119222	EU552153	-	-
	Ppf47	GU586851	-	-
*Pyrenochaeta parasitica*	CBS 451.73	AF525676	GQ387556	GQ387617
	CBS 218.77	-	GQ387557	GQ387618
*Pyrenochaeta quercina*	CBS 115095	*	GQ387558	GQ387619
	CBS 297.74	*	GQ387559	EU754177
*Pyrenochaeta unguis-hominis*	CBS 112.79	*	GQ387561	GQ387622
	CBS 378.92	EU930010	GQ387560	GQ387621
	CBS 111112	*	GQ387562	GQ387623
*Setophoma terrestris*	CBS 335.29	KF251246	GQ387526	GQ387587
*Chaetosphaeronema hispidulum*	CBS 216.75	KF251148	EU754045	EU754144

*Sequences obtained from Q-bank database.

### Gene prediction and annotation of assembled UM 256 genome

The putative genes of UM 256 were predicted from the UM 256 genome sequence using GeneMark-ES version 2.3 [16]. Functional annotations of the coding sequences (CDSs) were initiated using BLAST search against the NCBI non-redundant protein and SwissProt databases. Identification of Kyoto Encyclopedia of Genes and Genomes (KEGG) metabolic pathways was performed using local BLAST2GO tools [17]. Classification of putative proteins were performed using EuKaryotic Orthologous Group (KOG) [18] and the protein domain families were matched to the Pfam database using InterProScan 5 [19]. The rRNAs and tRNAs were predicted using RNAmmer v1.2 [20] and tRNAscan-SE v1.3.1 [21], respectively. The putative transposable elements were identified using Transposon-PSI (http://transposonpsi.sourceforge.net).

Functional annotation of Carbohydrate-Active enZymes (CAZymes) and peptidases were carried out by subjecting the predicted protein models to the database for automated Carbohydrate-active enzyme Annotation (dbCAN) [22] and MEROPS [23] databases, respectively. Prediction of signal peptide/non-signal peptide and cleavage sites of the secreted proteins were conducted by using SignalP version 4.1 [24]. The secreted proteins without transmembrane (TM) domains and those with single transmembrane present at the N-terminal 40 amino acids for secretion signal were selected. The presence of TM domains were identified by TMHMM version 2.0 [25]. The secondary metabolite biosynthesis backbone genes and clusters that present in the genome were analyzed using web-based SMURF (www.jcvi.org/smurf/) [26]. The gene sequences with an e-value threshold ≤1e-5, with the identity exceeding 50% and subject coverage more than 70% were selected and assigned as the predicted gene annotation. Mating type region was analyzed and retrieved from the sequence genome using the Artemis version 12.0 sequence viewer [27].

### Orthologous genes and comparative genomic analysis

The protein sequences of publicly available Dothideomycetes, Sordariomycetes, and Eurotiomycetes genomes were obtained from different databases (Table A in S1 File) to determine the orthologous genes in UM 256. The OrthoMCL version 2.02 [28] was used to analyze the protein sequences clustering (≥33 amino acids) for UM 256 and the 23 reference genomes by all-against-all BLASTp searches of all proteins. Orthologs were recognized by the reciprocal best blast hits from the distinct genomes.

### Validation of putative antifungal resistance genes

The culture of UM 256 was grown on SDA at 30°C for 12 days. The mycelium of the fungus was harvested and DNA extraction was performed according to the manufacturer’s instructions in ZR Fungal/ Bacterial DNA MiniPrep handbook (Zymo Research, USA). PCR was then carried out in a 25 μL reaction volume containing 5 μL of the extracted DNA, 0.2 μM of each primer, and 1× Go Taq Green Master Mix (Promega Corporation, USA). The primers used for amplification were shown in Table 2 and the PCR parameters consist of an initial denaturation at 95°C for 5 min, followed by 30 cycles of denaturation at 95°C for 30 seconds, annealing at 58°C for 30 seconds, extension at 72°C for 1 min to 6 min and final extension at 72°C for 7 min. The PCR products were then electrophoresed in 1% (w/v) agarose gel at 90V for 30 min. Subsequently, PCR products were purified and then sent for Sanger sequencing (1^st^ Base Laboratories, Malaysia).

**Table 2:** Primers and gene size (bp) of antifungal resistance genes that used for PCR amplification.

Genes	Primers	Sequences 5’ to 3’	Gene size (bp)
ERG11_A	11225F	ATGGGCGTCCTCGCTCAC	1682
	11225R	CTACGCCTTCTCCCTCCTT	
ERG11_B	2977F	ATGCCCTCACCTCTCAACC	444
	2977R	TCATCCCCTGTGTGCCTTG	
ERG11_C	2978F	ATGGCTCAGATCACCATTTTC	1005
	2978R	CTACGTGCTCTCCGCACG	
CDR1_A	9423F	ATGTGGGGAAACACCATCC	5177
	9423R	TTACACCGCCTCTTTCTTCC	
CDR1_B	5932F	ATGAGTTTGGTCGGCAATTTC	4820
	5932R	TTACACTTTCTCTGATATCTCG	
CDR1_C	11463F	ATGGGCTTCAAAGAGAGTGC	5003
	11463R	CTACGTGCTCTTCGTCTTC	
CDR1_D	3801F	ATGGCCTCAGACGAGCAAC	5396
	3801R	CTACGCTGCATCACCACTTT	
CDR2_A	3553F	ATGGCGAAGCCCACCGAG	4538
	3553R	TCAAGCCTTCGCTGCCTTTT	
CDR2_B	5895F	ATGGACAACGCGAACCATG	4782
	5985R	TCATCGTCGGTTAAAGTTCAG	
MDR1_A	8687F	ATGACTACCTCCGACGAGG	4464
	8687R	TTATCGATCCAAAGCTTGAGC	
MDR1_B	11980F	ATGGAGAACAAGCCCTCAG	4100
	11980R	TTATTGTGGTGCACCCATCG	
MDR2_A	7395F	ATGGGTGTGGAAGCACTCC	1468
	7395R	CTACTTGACCATCTTCTTCTC	
MDR2_B	8619F	ATGGAGCCGCCACGGTCA	4464
	8619R	TTATGTGGGTATACTCTGATC	

## Results and Discussion

### Morphology and Molecular Identification

**Fig 1:**
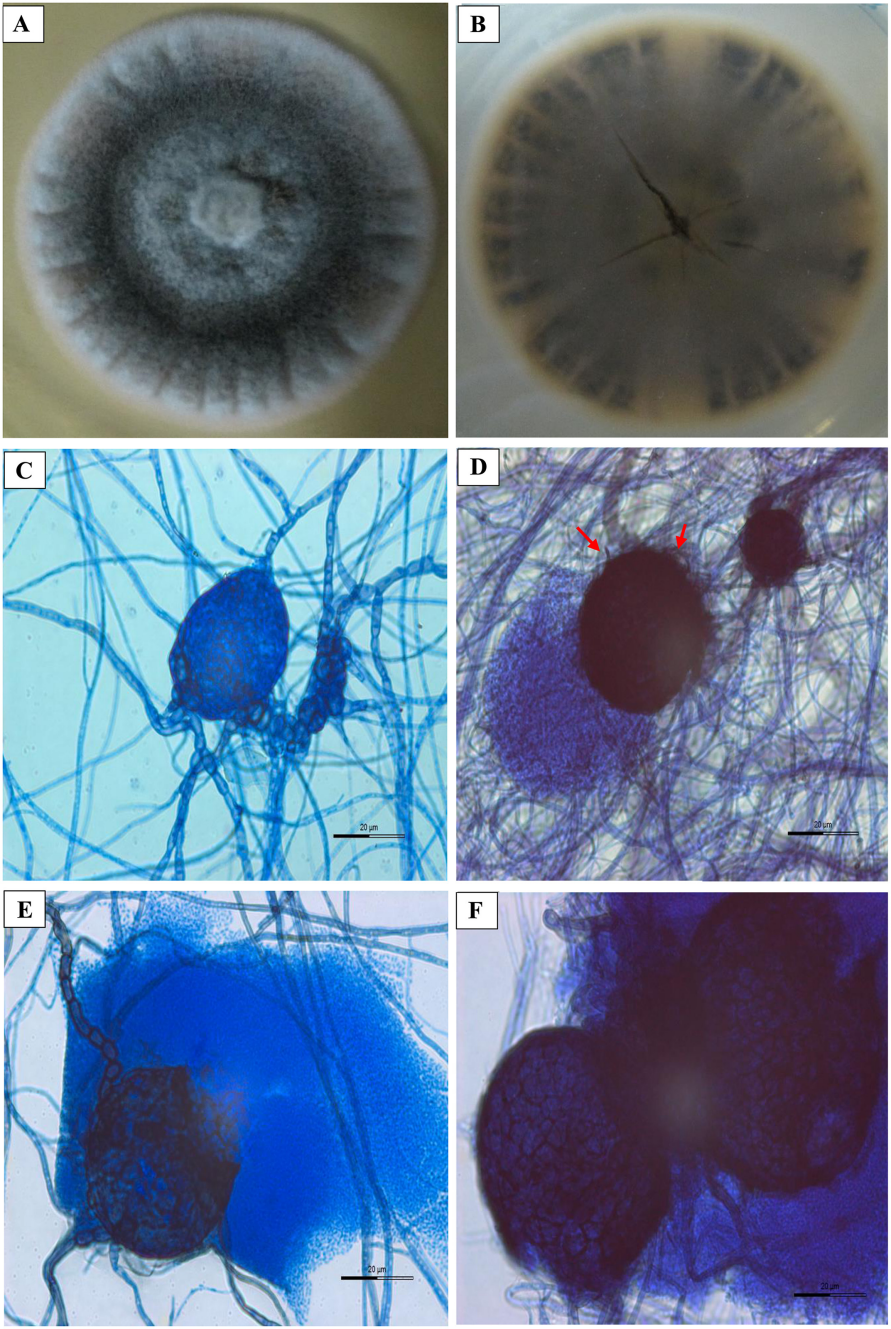
Colonial characteristic and microscopic morphology of *P*. *unguis-hominis* UM 256. Colonial morphology (A) front and (B) reverse of *P*. *unguis-hominis* UM 256 on SDA after 14-day incubation. In microscopic view, UM 256 showed (C) pycnidia, (D) mature pycnidia with short septate setae (arrow), (E) mature pycnidia and conidia, and (F) pycnidia wall with textura angularis (630× magnification, bars 20 μm).

*Pyrenochaeta* species and *Phoma* species share overlapping characteristics of conidiogenesis and setose pycnidia [1, 29]. In this study, the clinical isolate was first misidentified as *Phoma* species based on its morphological features. The UM 256 colony on SDA was flat, appeared from woolly to cottony with rough and rugose surface. The surface of the colony was firstly dark green and become dark grey with white periphery after being cultured for 14 days (Fig 1A). At the reverse, the colony is dark brown (Fig 1B). The isolate is a slow-growing fungus. The diameter of the colony was 5.3 cm (0.38cm per day) after 14 days incubation. Microscopic examination showed that the pycnidia (40 μm × 50 μm) of the fungus were solitary and globose or flask-shaped (Fig 1C). The pycnidia consist of several short septate setae, with phoma like conidiogenous cells (Fig 1D). A thickened pycnidia wall with textura angularis was observed (Fig 1E and 1F). Conidia were in single or clustered, curved or short cylindrical (Fig 1D, 1E and 1F).

**Fig 2:**
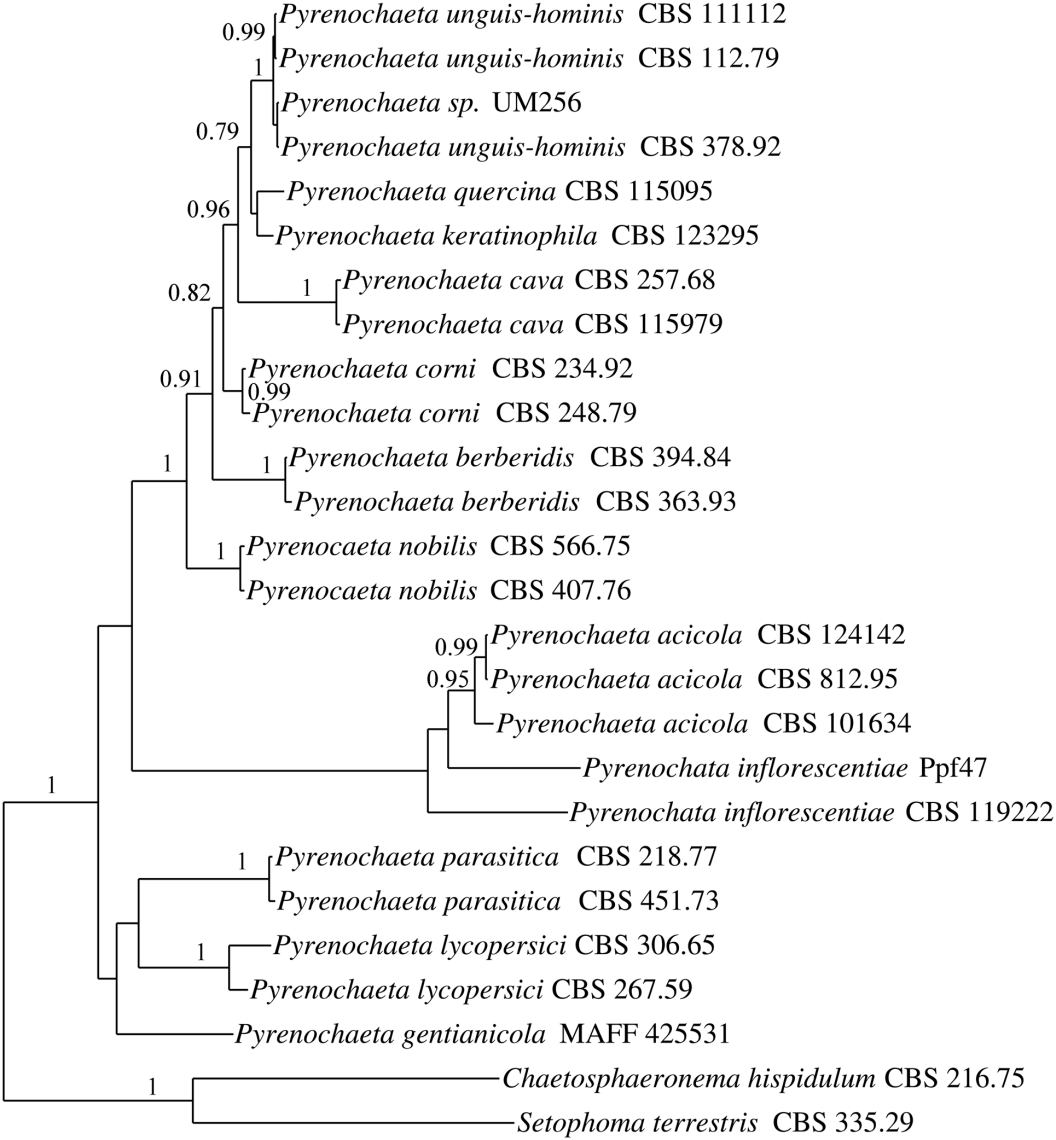
Bayesian phylogenetic tree of *Pyrenochaeta* sp. based on the combined genes of ITS, SSU and LSU sequenced data. The phylogenetic tree were constructed with 12 *Pyrenochaeta* species. The tree is rooted with *C*. *hispidulum* and *S*. *terrestris* as outgroup. The numbers on the nodes indicate Bayesian posterior probabilities.

Multilocus phylogenetic analysis of ITS, LSU, and SSU regions has been used to identify and differentiate *Pyrenochaeta* species from closely related *Phoma* species [1, 29]. In this study, multilocus phylogeny analysis showed that UM 256 was grouped together with three other *P*. *unguis-hominis* strains and it is tightly clustered together with *P*. *unguis-hominis* CBS 378.92 (Fig 2). The UM 256 isolate was thus confirmed as *P*. *unguis-hominis*.

### Gene prediction and annotation

The Roche 454 GS FLX+ and Roche 454 GS Junior sequencing systems were used to generate sequence reads for *P*. *unguis-hominis* UM 256. The sequenced reads were assembled using GS *de novo* Assembler version 2.70 (Table B in S1 File) [10]. The total assembly size of *P*. *unguis-hominis* UM 256 is 35.5 Mb. The *P*. *unguis-hominis* UM 256 genome contains a total of 12,545 putative coding DNA sequences (CDS) with an average gene length of 1,517 bp. Gene density of protein coding genes is 3.18/10 kb. Although the genome size of *P*. *unguis-hominis* UM 256 is smaller than the *P*. *lycopersici* genome (54.9 Mb) [2], the total number of predicted genes is lower but gene density is comparable to the *P*. *lycopersici* (17,000 genes and 3.09 genes/10 kb). In contrast, the *P*. *unguis-hominis* UM 256 genome size is larger as compared to *Pyrenochaeta berberidis* (sexual morph formally known as *Cucurbitaria berberidis*) genome (32.91 Mb) [30], however, *P*. *berberidis* contains a higher number of predicted genes (29,302 genes) and gene density (8.9 genes/10 kb). Of the 12,545 predicted gene models in UM 256, 11,847 and 7,753 coding sequences were annotated based on the proteins in NCBI non-redundant and SwissProt databases, respectively. Of the hypothetical proteins 8,411 were based on the top hit of the BLAST result against the NCBI non-redundant database. A total of 121 tRNAs and 33 rRNAs (22 8S, five 18S, and six 28S) were identified in *P*. *unguis-hominis* UM 256 genome.

**Fig 3:**
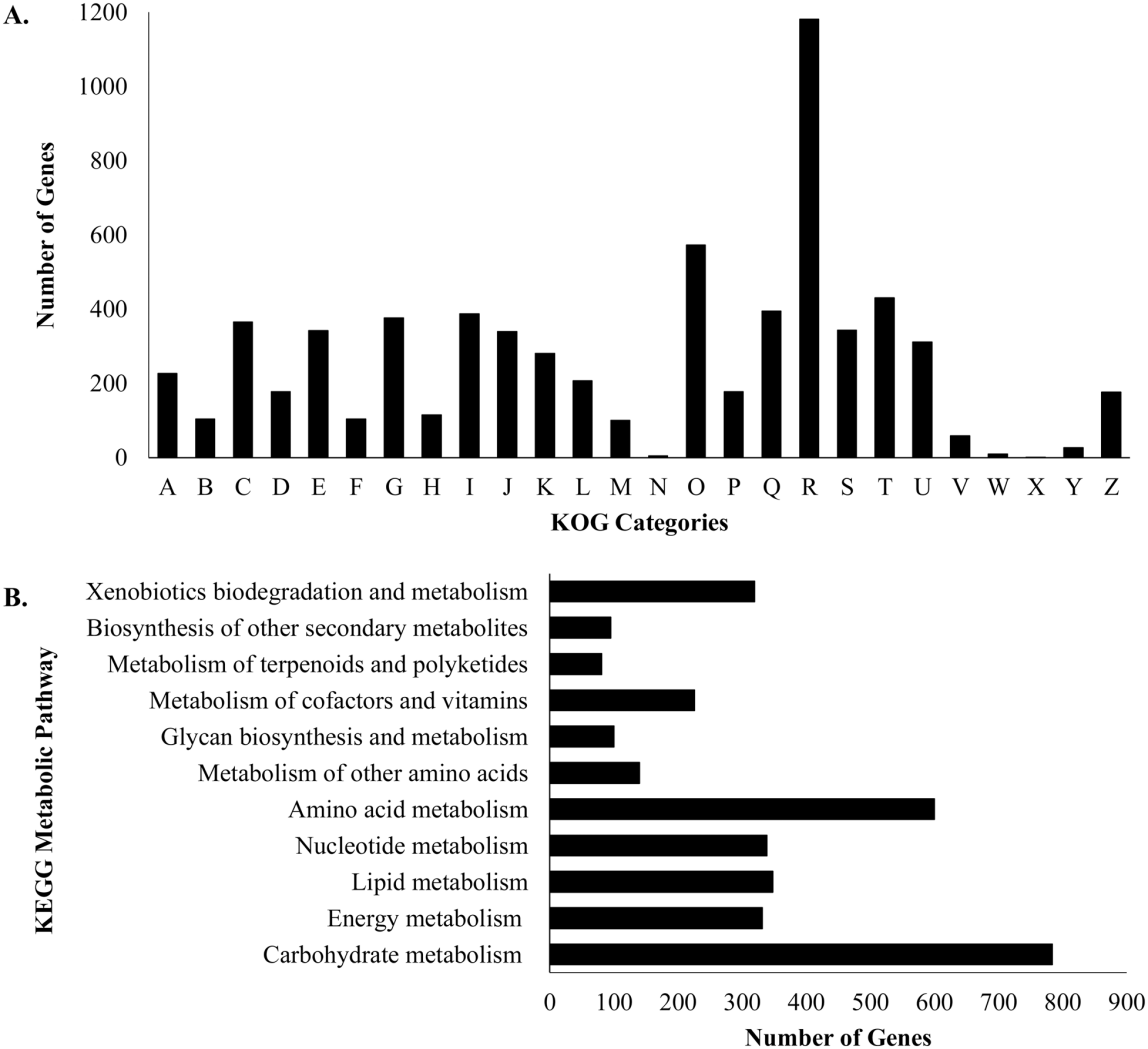
KOG and KEGG classifications of proteins in *P*. *unguis-hominis* UM 256. (A) KOG class annotation distribution of *P*. *unguis-hominis* UM 256 genome. A: RNA processing and modification; B: Chromatin structure and dynamics; C: Energy production and conversion; D: Cell cycle control, cell division, chromosome partitioning; E: Amino acid transport and metabolism; F: Nucleotide transport and metabolism; G: Carbohydrate transport and metabolism; H: Coenzyme transport and metabolism; I: Lipid transport and metabolism; J: Translation, ribosomal structure and biogenesis; K: Transcription; L: Replication, recombination and repair; M: Cell wall/membrane/envelope biogenesis; N: Cell motility; O: Post-translational modification, protein turnover, chaperones; P: Inorganic ion transport and metabolism; Q: Secondary metabolites biosynthesis, transport and catabolism; R: General function prediction only; S: Function unknown; T: Signal transduction mechanisms; U: Intracellular trafficking, secretion, and vesicular transport; V: Defense mechanisms; W: Extracellular structures; X: Unnamed protein and Z: Cytoskeleton. (B) Distribution of predicted proteins from *P*. *unguis-hominis* UM 256 genome that involved in metabolic pathway by KEGG database.

The UM 256 genome was mapped to KOG and KEGG databases to further characterize the predicted proteins. There are 6,813 proteins assigned to 26 different KOG categories (Fig 3A). Among all of the categories, the “General functions prediction only” category [R] has the most number of annotated genes (1,181), showing that the predicted proteins were not assigned to a specific group. This followed by the top five categories in the KOG group, including the category [O] “Posttranslational modification, protein turnover, chaperones” (573 genes), [T] “Signal transduction mechanisms” (430 genes), [Q] “Secondary metabolites biosynthesis, transport and catabolism” (395 genes), [I] “Lipid transport and metabolism” (388 genes), and [G] “Carbohydrate transport and metabolism” (376 genes). In this finding, it shows that most of the genes are involved in category O. In class [O], there were 121 genes annotated involved in ubiquitination, including ubiquitin activating enzyme (E1), ubiquitin conjugating enzyme (E2), and ubiquitin ligase (E3). These enzymes are responsible for the activation of ubiquitin and regulate the cellular processes in fungi, including cell growth, adaptation and development [31, 32]. These predicted enzymes might contribute to the ability of this fungus to survive in and adapt to adverse environments.

In the annotation of genes by KEGG, 1,337 predicted proteins were mapped to 11 KEGG metabolism pathways. Among the mapped pathways, carbohydrate metabolism (784 genes), amino acid metabolism (600 genes), lipid metabolism (348 genes), nucleotide metabolism (339 genes) and energy metabolism (332 genes) are the top five metabolic pathways (Fig 3B). Different mechanisms are involved in the carbohydrate metabolism such as glycolysis, gluconeogenesis, and citrate cycle (TCA) that provide carbon source as the nutrient for hyphal growth, sporulation, virulence and to maintain the cellular activities of the fungus [33, 34]. This result showed that large amounts of genes involved in carbohydrate metabolism allow UM 256 to use diverse sources for fungal growth.

### Transposable elements

In fungi, transposable elements (TEs) play an important role in speciation and adaptation [35]. They were shown to accelerate the genes evolution that affect the pathogenicity and host range [35]. A total of 0.34% (121,484 bases) in the assembled genome of UM 256 were identified as TEs, with 108 (0.28% of assembled genome) and 44 (0.06% of assembled genome) of class I TEs and class II DNA transposons, respectively (Table 3). Class I TEs, also known as retrotransposons that transpose by the reserve transcription of an RNA intermediate [36]. This class of TEs is subdivided into long terminal repeats (LTRs) and non-LTRs. Class II elements transpose directly via a DNA form by “cut and paste” mechanism [36]. Class II elements are divided in two subclass, including short inverted terminal repeats (ITRs) (subclass 1) and others with ITRs of variable length (subclass 2) [37]. In our finding, two LTRs families: Copia (15 copies) and Gypsy (68 copies) were identified *in P*. *unguis-hominis* UM 256, with Gypsy being the most abundant as previously described for most fungi [38]. The predominant Gypsy elements in filamentous fungi have been proposed as a unique DNA signature for strain identification [39]. LINE elements are non-LTR that originally found in mammal and have been reported to inactivates repeated DNAs occurs in a fungus by Repeat Induced Point mutation (RIP) mechanism [39]. A total of 0.02% (9 copies) LINE elements were identified in the assembled genome of UM 256. With regard to class II elements of UM 256, five families (cacta, hAT, mariner, mariner_ant1, MuDR_A_B) were grouped to the ITR while helitronORF was grouped to subclass 2. A total of 0.05% (23 copies) mariner elements were identified in UM 256 genome. Mariner elements are widely spread in nature and can be divided into different subfamilies including Tc1, Ant1 and pogo. Mariner_Ant1 was found in UM 256 and has been reported to involve in mobile and carry genomic sequences [36]. HelitronORF was the only subclass 2 elements that identified in 0.01% of the genome assembled. These elements are able to capture and amplify gene fragment by rolling-circle transposition mechanism [40]. In general, UM 256 contains small number of TEs in the genome, with more class I elements compared to class II elements. The abundance and distribution of the TEs might cause by the horizontal transfer, self-regulation transposition, and inactivation of repeat sequences [41].

**Table 3:** Transposable elements predicted in *P*. *unguis-hominis* UM 256.

Class	Family Name	Total Number	Total Bases	Genome assembled (%)
I	DDE_1	16	11,849	0.03
	Gypsy	68	74,257	0.21
	LINE	9	5,292	0.02
	TY1_Copia	15	6,129	0.02
II	Cacta	9	936	0.00
	hAT	3	1,362	0.00
	helitronORF	3	1,983	0.01
	Mariner	23	17,591	0.05
	Mariner_ant1	2	598	0.00
	MuDR_A_B	4	1,487	0.00
Total		152	121,484	0.34

### Phylogenomic analysis

**Fig 4:**
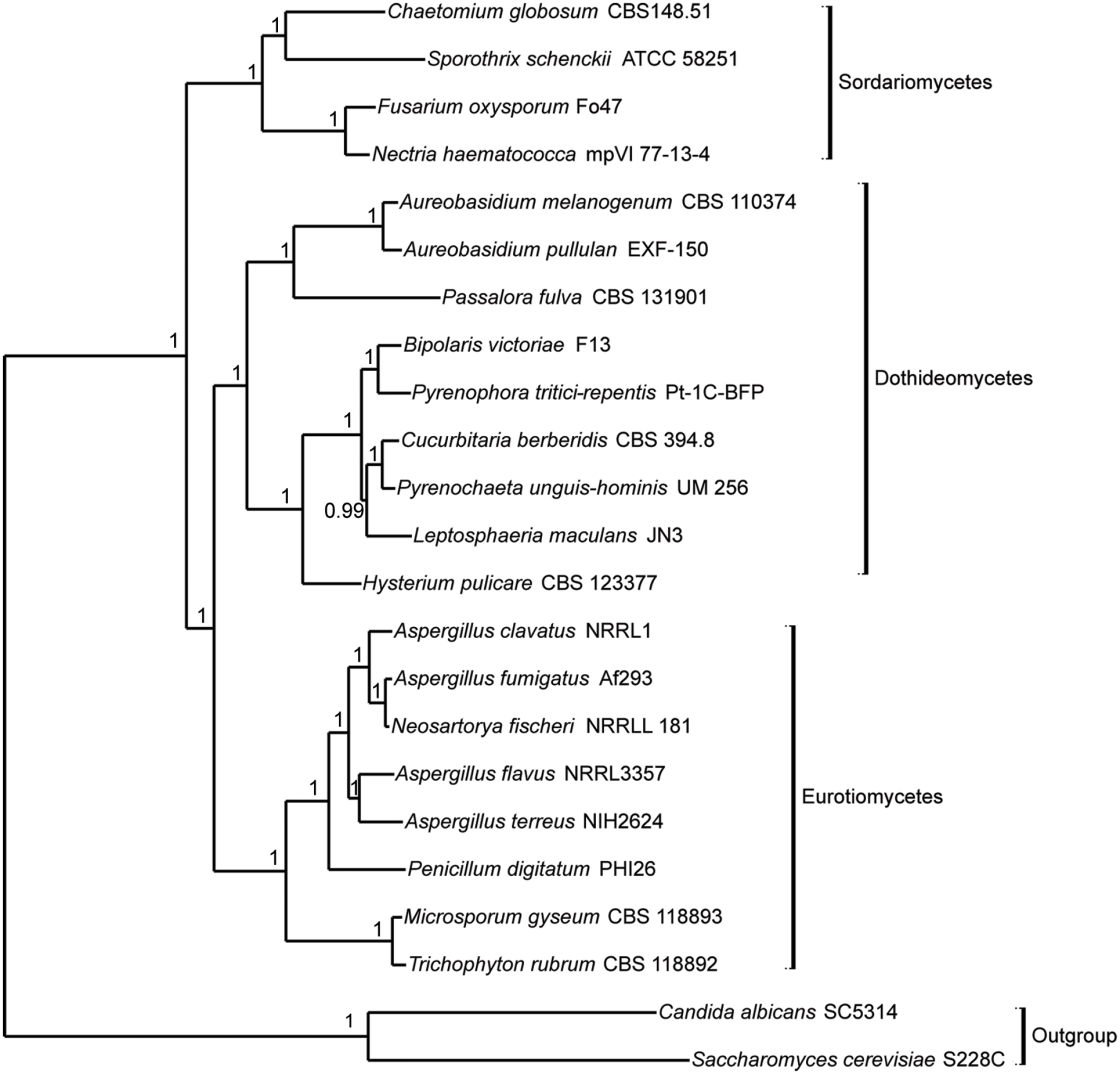
Phylogenomic analysis of *P*. *unguis-hominis* UM 256. The phylogenomic tree was constructed with total of 23 fungi including four from Sordariomycetes, nine from Dothideomycetes, eight from Eurotiomycetes and two outgroups from the Saccharomycetes (*C*. *albicans* and *S*. *cerevisiae*).

In the phylogenomic analysis, 23 fungal genomes encompassing two outgroups (*Candida albicans* and *Saccharomyces cerevisiae*) belonging to Saccharomycetes and UM 256 isolates was included in the analysis (Fig 4). A total of 266,057 proteins from selected species were subjected to all-against-all BLASTP and the proteins were clustered into 24,909 orthologous clusters where 42 single-copy orthologous genes were determined. These fungi were categorized into four classes, including Sordariomycetes (four species), Dothideomycetes (nine species), Eurotiomycetes (eight species) and Saccharomycetes (two species). UM 256 is clustered within the Dothideomycetes and grouped together with *Bipolaris vitoriae* F13, *Pyrenophora tritici-repentis* Pt-1C-BFP, *C*. *berberidis* CBS 394.8, and *Leptosphaeria maculans* JN3, which are belonging to the order of Pleosporales. In the same clade, UM 256 formed a monophyletic group with *C*. *berberidis* CBS 394.8.

### Gene families

A total of 24,909 gene family clusters were generated from 23 selected fungi, 14 genes families were specifically shared among the Dothideomycetes fungi and 404 gene family clusters were only shared between UM 256 and *C*. *berberidis* CBS 394.8 (Table C in S1 File). Among these 404 shared gene families, 269 are without annotation in the database, six with unknown protein functions, and 129 with known function (Table C in S1 File). The most abundant gene family clusters that were shared by both UM 256 and *C*. *berberidis* CBS 394.8 were heterokaryon incompatibility genes (16 clusters), followed by major facilitator superfamily domain (six clusters) and protein kinase domain (five clusters). These clusters are likely to be involved in adaptation, cellular response and interaction with the host as the heterokaryon incompatibility proteins are responsible in vegetative reproduction, producing viable heterokaryons necessary for fungal adaptation to environment as well as the host defence mechanism [42], the major facilitator superfamily transporters function to export secondary metabolites, host-derived antimicrobial compound and also involved in drug efflux systems [43–45], and protein kinases act as the mediators of fungal proliferation and development as well as signal transduction for reproduction [46].

The specific shared gene family clusters between UM 256 and *C*. *berberidis* CBS 394.8 associated with plant infection were also identified. These include genes encoding CFEM domain (Pyreno 23152), pectin lyase fold (Pyreno 23554), and rhamnogalacturonan lyase (Pyreno 23112). CFEM domain located at the fungal cell membrane serves as the cell surface receptors or adhesion molecules in host interactions, and might play a role in pathogenesis [47]. The pectin lyase fold and rhamnogalacturonan lyase are responsible for cleaving the bonds between homogalacturonan and the rhamnogalacturonan-I backbone during pectin degradation [48]. As no study was conducted on *P*. *unguis-hominis* in plant cell wall degradation, the presence of gene encoding pectin lyase and rhamnogalacturonanan lyase might at least suggest the ability of UM 256 to produce pectin enzymes for pectin degradation.

### Sexual reproduction in *P*. *unguis hominis* UM 256

**Fig 5:**
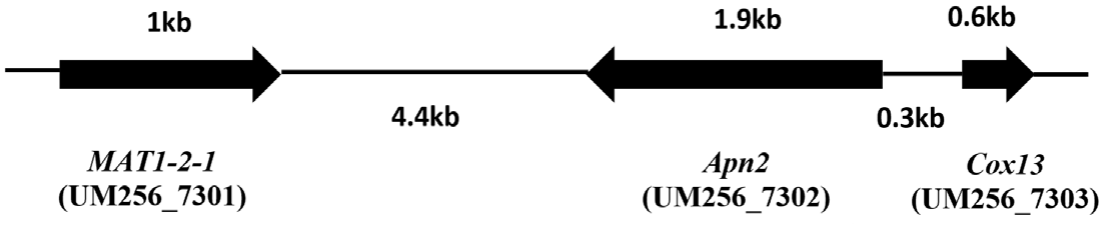
*MAT1-2-1* gene of *P*. *unguis-hominis* UM256. Genes constituting the MAT locus: *MAT1-2-1* (UM256_7301), DNA lyase, *Apn2* (UM 256_7302) and cytochrome C oxidase subunit Vla, *Cox13* (UM256_7303).

In the genus of *Pyrenochaeta*, little is known of sexual reproduction. The only *Pyrenochaeta* species reported with asexual state is *P*. *berberidis* [1]. Sexual reproduction in fungi was reported to occur in two distinct manners: homothallic (self-fertile) or heterothallic (requiring a partner) [49]. In this study, we manage to reveal several putative genes for mating, fruiting body development, and meiosis in UM 256 genome (Table D in S1 File). A single mating type encoding a high mobility group (HMG)-domain containing *MAT1-2-1* gene (UM256_7301; 53% identical) was identified in this genome. Besides, a DNA lyase *APN2* (UM256_7302) and a cytochrome c oxidase subunit V1a *Cox13* (UM256_7303) were also found flanking to the *MAT1-2-1* gene (Fig 5; Table D in S1 File). The configuration of this mating locus with the presence of these two genes has been reported before [50]. As only *MAT1-2-1* gene was identified, it suggests that UM 256 could be a heterothallic or asexual fungus.

During sexual reproduction, heterothallic fungi stimulate and respond to the pheromone secreted by a strain of opposite mating type through a pheromone response pathway [51]. In UM 256, the major components involved in pheromone response pathway were identified (Table D in S1 File). This includes the pheromone receptor, PreB (UM256_9138, 55.4%) and PreA (UM256_2801, 56.5%) which are responsible to bind with the pheromone were predicted [52]. Besides, G-protein α subunit (Gpa1) (UM256_360, 99.7%), G-protein β subunit (sfaD; UM256_5190, 83%) and a G-protein γ subunit (Ste18; UM256_7215, 96.9%) which transmit the signal to a scaffold protein (Ste5) and a p21-activated protein kinase (Ste20) were identified in UM 256 genome.[52] Ste20 (UM256_10447, 76.7%) was predicted but Ste5 was not found in UM 256 genome. However, the Ste11-Ste7-Fus3/Kss1 (UM256_5614, 88.9%; UM256_2176, 63% and UM256_5408, 96.3%) cascade which is activated by Ste20 was identified in UM 256. Besides, a transcription factor (SteA) (UM256_6395, 88.2%) homolog of Ste12 which responsible to regulate the mating process was also predicted in UM 256. Overall, the presence of the mating process genes in UM 256 would enable this fungus to response to the pheromone during the sexual reproduction.

### Carbohydrate active enzymes (CAZymes)

**Fig 6:**
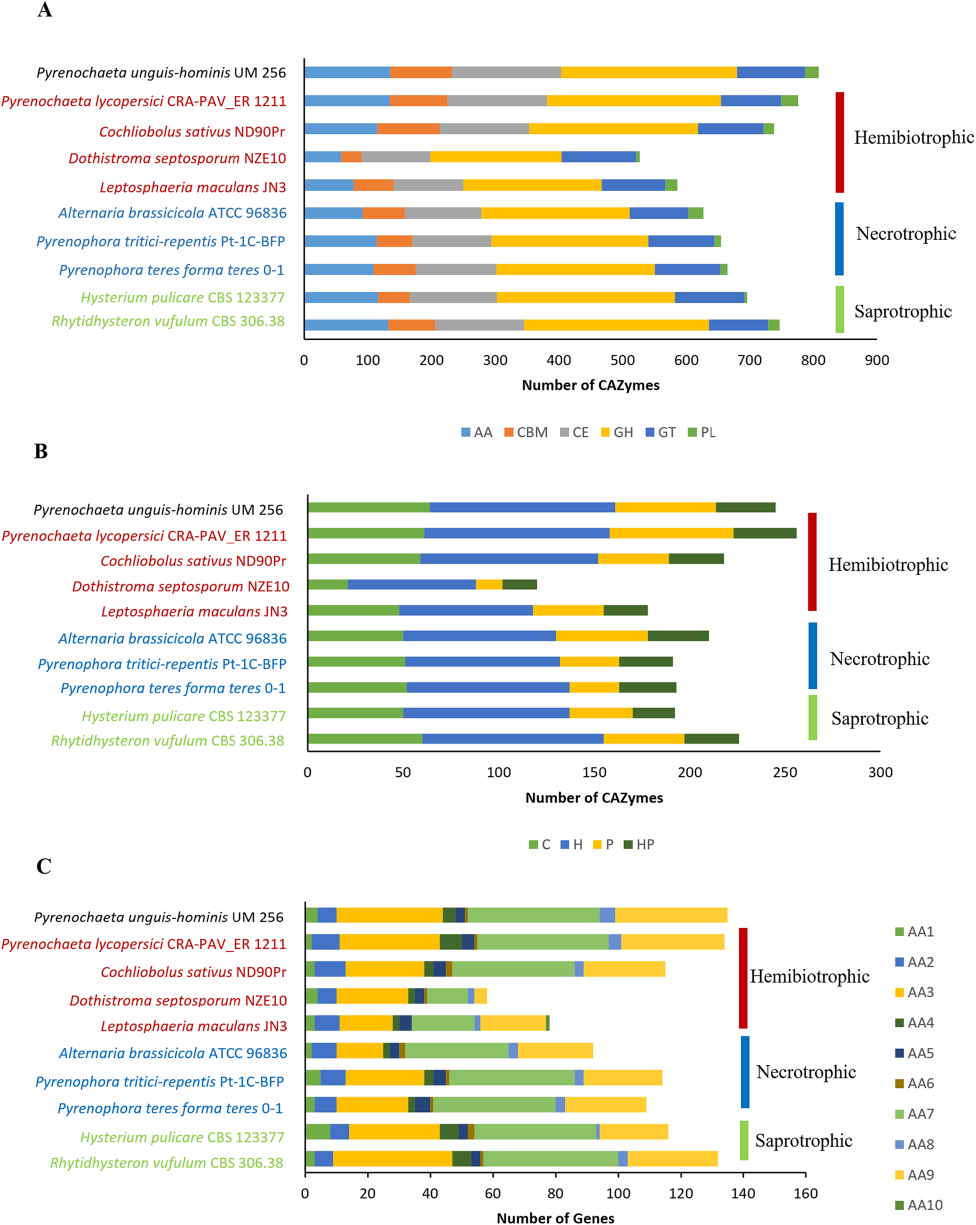
CAZymes class distribution of *P*. *unguis-hominis* and others Dothideomycetes. (A) Distribution of each of the CAZymes family among *P*. *unguis-hominis* UM 256 and other Dohideomycetes fungi. (B) Distribution of CAZymes family involved in plant cell wall degradation. (C) Auxiliary group (AA) of CAZymes distribution among *P*. *unguis-hominis* UM 256 and others Dothideomycetes fungi. AA, auxiliary activities; CBM, carbohydrate-binding modules; CE, carbohydrate esterases; GH, glycoside hydrolases; GT, glycosyltransferases; PL, polysaccharide lyases. C, cellulose; H, Hemicellulose; P, pectin; HP, enzymes that degrade hemicellulose of pectin side chain.

Fungal CAZymes are important components that degrade the plant cell wall into simple monomers to serve as nutrient for fungal growth [53]. A total of 808 putative CAZymes were identified in UM 256 genome, including 277 glycoside hydrolases (GH), 171 carbohydrate esterases (CE), 135 auxiliary activities (AA), 107 glycosyltransferases (GT), 97 carbohydrate-binding modules (CBM) and 21 polyssaccharide lyases (PL) (Fig 6A). The putative CAZymes in UM 256 were then compared to other nine Dothideomycetes fungi to further gain insight into its lifestyle. The result showed that UM 256 has the highest CAZymes compared to necrotrophic, hemibiotrophic, and saprophytic fungi (Fig 6A; Table E in S1 File).

A comparison analysis was also done based on the substrate specificity of CAZymes that are involved in plant cell wall degradation, i.e cellulose, hemicellulose and pectin. Among these fungi, UM 256 contains the highest number of cellulose degrading enzymes (64 putative CAZyme), but its ratio of cellulose versus plant cell wall degrading genes was comparable with other pathogenic fungi (Fig 6B; Table F in S1 File). Cellulose degradation requires collaboration of several enzymes in GH class [53]. There were different GH families identified in UM 256, including cellobiohydrolase (GH6 and GH7), endoglucanase (GH12, GH45 and GH61), and β-glucosidase (GH1 and GH3). These enzymes are involved in the breakdown of crystalline regions in the cellulose and hydrolyzing the cellobiose to glucose [54, 55]. Besides, a total of 97 putative CAZymes genes (39.6%) in UM 256 were predicted to be involved in hemicellulose degradation. The amount of predicted genes are comparable to others Dothideomycetes fungi (Fig 6B; Table F in S1 File). A total of 16 CAZymes families, GH10, GH11, GH26, GH27, GH29, GH31, GH35, GH36, GH39, GH67, CE1, CE2, CE3, CE5, CE15 and CE16 involved in the hemicellulose degradation were reported in previously [56]. In UM 256 especially large numbers of enzymes of CE1 family are found but it lacks members of the GH29 and GH39 CAZymes families altogether. The CE1 family in UM 256 includes genes encoding for acetyl xylan esterase, feruloyl esterase and S-formylglutathione hydrolase which enables the acetylation of hemicellulose residues [57]. The number of modules involved in pectin degradation in UM 256 (53 genes; 21.6%) is higher than in the other Dothideomycetes fungi, but is slightly lower than in *P*. *lycopersici* (Fig 6B; Table F in S1 File). A total of 11 CAZymes families, including GH28, GH78, GH88, GH95, GH105, GH115, PL1, PL3, PL4, PL9, and CE8 were found to be involved in the pectin degradation of UM 256. The pectin matrix in the plant cell wall consists of highly complex polysaccharides including homogalacturonan (HG), rhamnogalacturonan-I (RGI), rhamnogalacturonan-II (RGII) [58]. In UM 256, the GH28 family contains exo-polygalacturonases and rhamnogalactoronases which are able to cleave the HG chain residues and rhamnose residue in RGI [48]. Besides, families GH78 and GH105 are also involved in the degradation of the RGI backbone specifically [59]. Moreover, families involved in pectin degradation, PL1, PL3, PL4 and CE8 were identified in UM 256. The pectin lyases from PL1 and PL3 are responsible for cleaving the bonds linked to the HG backbone, whereas PL4 (rhamnogalacturonate lyase) breaks the bonds linked to the RGI backbone [48]. The pectin methylesterase from CE8 (pectin methylesterase) removes the methyl groups in HG [60].

Furthermore, we also performed a comparative analysis on the AA family between UM 256 and other Dothideomycetes fungi. It was shown that UM 256 has the highest number of AA family genes (135 genes) (Fig 6C) and was comparable to *P*. *lycopersici* (17.3%) and *Pyrenophora tritici-repentis* (17.4%) in the ratio of AA family versus total number of CAZymes (Table E in S1 File). Most of the AA families were found in the genome except for the AA10 and AA11 families. AA10 has been reported to be predominantly found in bacteria and is less common in eukaryotes whereas AA11 enzymes that function to cleave chitin are mostly found in dermatophytic ascomycetes [61, 62]. In particular, AA3, AA7 and AA9 genes were prominently present, with 34, 42 and 36 CAZymes. These numbers were higher as compared to the other Dothideomycetes fungi, with the exception of AA3 and AA7 of *Rhytidhysteron vufulum* (Table G in S1 File). However, in the ratio of each AA families versus total number of AA group, UM 256 has lower AA3 (25.1%) and AA7 (31.1%) compared to others Dothideomycetes fungi. The ratio also showed that AA8 (3.7%) in UM 256 was the highest among others fungi and AA9 (26.7%) was comparable to *Leptosphaeria maculans* (26.9%). The family AA3 (glucose-methanol-choline oxidoreductases family) consists of cellobiose dehydrogenases, aryl-alcohol oxidase, glucose oxidase, and alcohol oxidase which play roles in cellulose, hemicellulose and lignin biodegradation [63]. Besides, gluco-oligosaccharide oxidase (AA7 family) and lytic polysaccharide monooxygenases (AA9 family) have been reported to oxidize the carbohydrates and cleave the glucose chain during cellulose degradation [63]. The AA9 members have been reported mainly in fungal genomes of fungal wood decayers [63].

Overall, UM 256 has a large potential capability to hydrolyze the polysaccharides and to degrade the plant cell wall for infection [2]. Besides, the CAZymes content in the genome of UM 256 exhibit more preference to cellulose and hemicellulose rather than pectin and the strain contains a large amount of AA family that able to breakdown lignin. This suggest that UM 256 is capable of vascular plant cell wall degradation.

### Secondary metabolism

In UM 256, 21 secondary metabolite backbone genes were identified with 18 of the genes found to be clustered. Of these genes, 11 are nonribosomal peptide synthases (NRPS) or NRPS-like, nine polyketide synthases (PKS) or PKS-like, and one dimethylallyl tryptophan synthase (DMAT) (Table H in S1 File).

Iron is an important element for most organisms, as it is required in the metabolism acts as a cofactor and catalyst in metabolic pathway [64, 65]. However, an overloaded of iron will cause cell damage via the Fenton reaction [65]. Therefore, iron regulation is needed to maintain the uptake and storage of iron such as by siderophore [66]. A siderophore is a small molecule that act as iron chelate to mediate the uptake of iron. The predicted putative genes that are involved in the siderophore biosynthesis identified in UM 256 are similar to the reported genes responsible in *Aspergilus fumigatus* siderophore biosynthesis [67]. The putative gene, nonribosomal peptide synthase 6 (NRPS6, UM256_3918) is similar to *A*. *fumigatus Sid D* with 43.7% identity and also, consists of a domain arrangement of A-T-C-T-T-C as the *A*. *fumigatus Sid D*. Besides, the genes encoding L-ornithine 5 monooxygenase (*Sid A*, UM256_3916, 35.5%), acyl-CoA N-acyltransferase (*Sid F*, UM256_3915, 44.4%) and ferrichrome siderophore peptidase synthetase (*Sid C*, UM256_47, 25.9%) encoding genes were predicted too. The putative *Sid A* (UM_3916) and *Sid F* (UM256_3915) genes were clustered together with *Sid D* (UM256_3918) (Table I in S1 File). In siderophore biosynthesis, *Sid A* and *Sid C* were reported to be involved in the synthesis of ferrichromes which are responsible for the intercellular hyphal and conidia iron storage [68]. On the other hand, *Sid A*, *Sid F*, *Sid D* and *Sid G* are involved in the production of triacetylfusarinine C (TAFC). TAFC has been shown to be a virulence factor in *A*. *fumigatus* [67]. However, in our analysis, *Sid G* which catalyzes the conversion of fusarinine C (FSC) to TAFC was not identified. Hence, TAFC might not be produced by this fungus. Nevertheless, lack of TAFC does not have much effect on the regulation of iron as FSC was reported capable to replace TAFC as a siderophore *in vivo* and *in vitro* [67]. Furthermore, a siderophore transporter (UM256_4069, 66.4%) which is homologous to the *MirB* gene of *A*. *nidulans* was also predicted in UM 256 [66]. Therefore, the predicted putative genes of UM 256 involved in siderophore production suggested that UM 256 is able to produce siderophores for its iron regulation.

Melanin play an important role in fungal protection against oxidants, high temperatures, UV irradiation and several other stress conditions. Most of the Dothideomycetes fungi produce DHN-melanin via the polyketide synthase pathway [69]. In UM 256, a PKS (UM 256_10683) that encodes the precursor of melanin pigment production was identified. It was annotated as conidial pigment biosynthesis polyketide synthase with 83.4% identity to the *A*. *alternata* PKS (*ALM*) [70]. The predicted PKS contains the domain arrangement of KS-AT-DH-ACP-ACP-TE which was similar to the PKS reported in melanin biosynthesis [71]. Besides, one 1,3,8-trihydroxynapthalene reductase gene (UM256_10680, 94.4%) homologous to the *BRM2* gene in *A*. *alternata* and a gene encoding transcription factor *Cmr1* (UM256_10681, 82.9%) were found in cluster with the PKS gene. Additionally, two scytalone dehydratase genes (UM256_246, 38.4%; UM256_3051, 87.2%) and one tetrahydroxynaphthalene reductase genes (UM 256_9046, 93.6%) which are involved in melanin biosynthesis were also predicted in UM 256 (Table J in S1 File). Thus, this suggests that the dark pigment of UM 256 is synthesized via DHN-melanin pathway.

### Peptidases involved in keratin degradation

Peptidases are enzymes that have the capability to digest protein and degrade host tissue [50, 72]. A total of 183 peptidases were identified in UM 256, of which, 48 are secreted peptidases and mostly are from serine peptidases and metallopeptidases (21 serine peptidases, 15 metallopeptidases, five aspartic peptidases, two cysteine peptidases and one glutamic peptidase) (Table K in S1 File). Secreted peptidases from metallopeptidase and serine peptidase families are reported to be involved in degradation of keratin [73, 74]. The fungalysin (M36) and subtilisin (S8A) families are endopeptidases reported to invade the epidermis of the host in dermatophytes [74–76]. In UM 256, there is one gene (UM256_2144) encoding the fungalysin with 79.6% identity to *A*. *fumigatus*. Besides, there are two subtilisin encoding genes similar to *Engyodontium album* PR1 (UM256_3877, 54.8%) and *S*. *cerevisiae* PRB1 (UM256_12659, 51.1%) (Table 4). On the others hand, one dipeptidyl peptidase IV (DppIV) (S9B), two dipeptidyl peptidase V (DppV) (S9C), two leucine aminopeptidase 1 (LAP1) (M28), seven metallocarboxypeptidase (M14) and eight carboxypeptidase (S10) encoding genes were predicted in UM 256. These exopeptidases are responsible for cleaving the peptide bond at the polypeptidase N- or C-terminal during degradation of keratin [74]. Moreover, DppIV, DppV and LAP1 of UM 256 are similar to the peptidases encoded by *Aspergillus* species. During keratin tissue degradation, Laps degrade peptides from the N-terminus until they reaches an X-Pro sequence. Complementary, DppIV removes these X-Pro sequences, and thus allowing Laps access to next residue [77, 78]. Furthermore, efficient keratin degradation requires large amount of secreted sulphite to cleave the disulphide bridges of keratin into cysteine and S-sulphocysteine, allowing the reduced proteins to become accessible for further digestion by various endo-exopeptidase [79]. Thus, the presence of sulphite transporter (SSU1) is important in keratin degradation [80]. A *SSU1* gene (UM256_209) in UM256, sharing 54.7% similarity to the *SSU1* gene in *Arthoderma benhamiae* was predicted (Table 4). Therefore, this suggest that UM 256 might have the capability to adhere and invade skin and nail.

**Table 4:** Peptidase and genes that involved in keratin degradation of *P*. *unguis-hominis* UM 256.

Peptidase / Transporter	Families	Gene ID	Description
**Endopeptidase**	M36	UM256_2144	Fungalysin
	S8A	UM256_3877	Subtilisin
		UM256_12659	
**Exopeptidase**	M14	UM256_664	Carboxypeptidase
		UM256_2577	
		UM256_4019	
		UM256_6089	
		UM256_8811	
		UM256_8899	
		UM256_10121	
	M28A	UM256_3201	Leucine aminopeptidase 1
		UM256_9784	
	S9B	UM256_4508	Dipeptidyl peptidase IV
	S9C	UM256_3549	Dipeptidyl peptidase V
		UM256_9888	
	S10	UM256_2488	Carboxypeptidase
		UM256_3808	
		UM256_4495	
		UM256_5706	
		UM256_6549	
		UM256_7383	
		UM256_7507	
		UM256_10480	
**Transporter**	SSU1	UM256_209	Sulphite transporter

### Antifungal resistance gene in *P*. *unguis-hominis* UM 256

Yew *et al*. (2014) reported that *P*. *unguis-hominis* UM 256 was resistant to azole drugs (fluconazole, itraconazole, posaconazole and voriconazole) and echinocandin (caspofungin) [9]. Thus, it is important to gain insight into the genetic basis of multidrug resistance in the isolate. The azole resistance mechanisms a well described in certain yeasts as well as filamentous fungi [81]. In this paper, a total of 14 antifungal resistance genes (*ERG11/CYP51*, *CDR1*, *CDR2*, *MDR1*, and *MDR2*) were amplified from the genomic DNA of UM 256 to validate the sequenced genomic data (S1 Fig). Out of the 14 antifungal resistance genes, 13 of the genes were successfully amplified.

The azole resistance can be caused by mutations or overexpression of lanosterol 14α-demethylase and/or upregulation of multidrug efflux pumps [82]. Lanosterol 14α-demethylase is the product of *ERG11/CYP51* gene which is the target of the azole drugs [82]. In this study, three putative *ERG11/CYP51* genes were predicted, including UM256_11225 (86.5% identical), UM256_2977 (66.2% identical) and UM256_2978 (62.9%) (Table L in S1 File). The sequences were compared with previously published ERG11 protein sequences of the azole drug resistant *C*. *albicans* (GenBank accession number X13296 and AF153846). The mutations at V61T, A107T and K119L mutations are associated with the azole drugs resistance in *C*. *albicans* as previously reported [83] are detected in UM256_11225 (V65T) and in UM256_2977 (A93G and K106G) (Table 5, S2A and S2B Fig). Furthermore, compelling reports showed that mutations detected in *ERG11/CYP51* genes were found in combination. As reported before, the decrease in the affinity for azole drugs is greater when the mutations are combined compared to single amino acid change [84]. Mutation such as D116E and E226D were found simultaneously with other mutations in many *C*. *albicans* isolates with fluconazole resistance, including D116E-V437I and D116E-K119L-E226D [84]. These combined mutations were identified in UM 256_11225 as D120E-V435A and D120E-K123A-E267S. In this study, we also detected several potential combined mutation site that studied by Ying *et al*.[84] in UM256_11225 and UM256_2978, including T230S-F447W, T38S-F253Y and I62M-V244G (Table 5, S2A and S2C Fig). As compelling reports showed that several mutations detected in *ERG11/CYP51* gene were found in combination [84], these combinations of mutation sites identified in gene UM256_11225 and UM256_2978 suggest its role in azole resistance.

**Table 5:** Amino acid substitution detected in *ERG 11/CYP51* genes of *P*. *unguis-hominis* UM 256.

Gene ID	Amino acid substitution in *C*. *albicans*	Amino acid substitution in *P*. *unguis-hominis* UM 256
**UM256_11225**	V61T	V65T
	D116E + V437I	D120E + V435A
	D116E, K119L + E266D	D120E, K123A + E267S
	T229A + F449S	T230S + F447W
**UM256_2978**	T229A + F449S	T38S + F253Y
	I253V + V437I	I62M + V244G
**UM256_2977**	A107T	A93G
	K119L	K106G

The multidrug efflux pumps that are involved in antifungal resistance are major facilitator superfamily (MFS) and ATP-binding cassette (ABC) transporters. MFS transporters contain two type of proton antiport, including drug: H^+^ antiport 1 (12 transmembrane spans) DHA1 family and drug: H^+^ antiport 2 (14 transmembrane spans) DHA2 family [85]. In our finding, fourteen DHA1 families and one DHA2 family were predicted in UM 256 (Table M in S1 File). ABC transporters encoded by *candida* multidrug resistance genes (*CDR1* and *CDR2*) and multidrug resistance genes (*MDR*1 and *MDR*2) play a major role in azole resistance [86]. In this study, a total of seven *CDR*1, two *CDR*2, eight *MDR*1 and four *MDR*2 were predicted in UM 256 (Table N in S1 File). Comparative gene families analysis revealed that UM 256 has a higher number of *CDR* and *MDR* genes than other fungi (Table O in S1 File). The presence of mutated genes and large number of drug transporters seem to correlate to the resistance of UM 256 to various azole drugs.

Unlike azole drug resistance mechanism, reduced susceptibility to echinocandins in fungi is attained by the amino acid substitution in Fks1 subunits [87]. Caspofungin resistance in UM 256 might be caused by the mutations in the Fks1 subunit (UM256_4224, 78%), in a conserved “hot-spot” region 5’-FLTLSLRDP-3’ (S3 Fig). Comparison of the subunit with Fks1 subunits from the caspofungin resistant *C*. *albicans* (GenBank accession number D88815) and *A*. *fumigatus* (GenBank accession number U79728) indicated the L637I and R638K in UM256_4224 might confer to caspofungin resistance in UM 256.

## Conclusions

In this study, we successfully identified and characterized UM 256 morphologically and molecularly. Through phylogenetic and phylogenomic analyses, we reveal that UM 256 belongs to *P*. *unguis-hominis* and is grouped under the order of Pleosporales, class of Dothideomycetes. We identified the *MAT1-2-1* type genes and other genes putatively involved in sexual reproduction suggesting that this fungus is able to reproduce sexually. Further analysis on this fungus also showed the presence of various genes involved in the carbohydrate and protein catabolism. This suggested that UM 256 has the capability to degrade plant cell wall, particularly in cellulose and lignin. Besides, the presence of keratin tissue degradation genes found in UM 256, suggests the potential of UM 256 to break down keratin tissue of the host, providing an opportunity for skin infection. Furthermore, UM 256 is enriched in putative PKS, NRPS, and DMAT genes. This isolate is able to synthesize siderophore and melanin. Interestingly, UM 256 was found to be resistant to most of the antifungal drugs, especially azole. In this work, putative antifungal resistance related genes were predicted in UM 256 including *MDR*, *CDR* and *ERG11/CYP51*, and we have successfully determined the presence of these antifungal genes in this genome. We hope that the study of *P*. *unguis-hominis* UM 256 characteristics and in-depth analysis of the genome content will provide an opportunity for a better understanding of its biology, enrich the current knowledge and contribute to future fungal research.

## Supporting Information

S1 Fig PCR amplification of azole resistance genes. **(A) *ERG11/CYP51* genes.** Lane 3, lane 5, lane 7 and lane 9 are the negative control. The PCR products for *ERG 11/ CYP51* genes (lane 4: UM256_11225, lane 5: UM256_2977, lane 8: UM256_2978, and lane 10: UM256_1421) showed targeted single band. **(B) *CDR1* genes.** Lane 2, lane 4, lane 6, lane 8 and lane 10 are the negative control. The PCR products for *CDR1* genes (lane 1: UM256_9423, lane 5: UM256_5932, lane 7: UM256_11463, and lane 9: UM256_3801) showed targeted single band. However, PCR products at lane 6 (UM256_7510) showed no band. **(C) *CDR2* genes.** Lane 1, and lane 3 are the negative control. The PCR products for *CDR2* genes (lane 2: UM256_3553 and lane 4: UM256_5895) showed targeted single band. **(D) *MDR1* genes.** Lane 1, and lane 3 are the negative control. The PCR products for *MDR1* genes (lane 2: UM256_8687, and lane 4: UM256_11980) showed targeted single band. **(E) *MDR2* genes**. Lane 2 and lane 4 are the negative control. The PCR products for *MDR2* genes (lane 1: UM256_7395, and lane 3: UM256_8619) showed targeted single band. The ladder used for (A) and (E) is 500 bp DNA ladder (iDNA Biotechnology, Malaysia) and the ladder used for (B), (C) and (D) is 1kb DNA ladder (Thermo scientific, US).
(PDF)

S2 Fig Alignment of *ERG11/CYP51* genes sequence of UM 256 with two *C*. *albicans* (GenBank accession number X13296 and AF153846). The point mutation site are shown (arrow) in *ERG*11 genes (A) UM256_11225 (B) UM256_2977 (C) UM256_2978.
(PDF)

S3 Fig Alignment of UM256_4224, *C*. *albicans* (GenBank D88815) and *A*. *fumigatus* (GenBank U79728). Point mutations was shown in FKS “hot-spot” 1 regions of UM256_4224, *C*. *albicans* and *A*. *fumigatus*.
(PDF)

S1 File **Table A in S1 File**. List of *P*. *unguis-hominis* UM 256 and 22 fungi under Dothideomycetes, Sordariomycetes, and Eurotiomycetes used for genome comparative analysis and phylogenomic tree construction. **Table B in S1 File**. Genome features of *P*. *unguis-hominis* UM 256. **Table C in S1 File**. Gene families share by both *P*. *unguis-hominis* UM 256 and *C*. *berberidis* CBS 394.8. **Table D in S1 File**. List of mating process genes, pheromone response genes, fruiting body development and meiosis specific genes in *P*. *unguis-hominis* UM 256. **Table E in S1 File**. CAZymes families in *P*. *unguis-hominis* UM 256 and other Dothideomycetes fungi. **Table F in S1 File**. Comparison of plant cell wall degrading and modifying CAZyme families in *P*. *unguis-hominis* UM 256 and other Dothideomycetes fungi. **Table G in S1 File**. Auxiliary activities of *P*. *unguis-hominis* UM 256 and others Dothideomycetes fungi. **Table H in S1 File**. Secondary metabolite backbone genes of *P*. *unguis-hominis* UM 256 predicted by SMURF. **Table I in S1 File**. Show the putative genes involved in siderophore biosynthesis of *P*. *unguis-hominis* UM 256 compared with *A*. *fumigatus*. **Table J in S1 File**. Melanin biosynthesis putative genes predicted in *P*. *unguis-hominis* UM 256. **Table K in S1 File**. Secreted peptidase of *P*. *unguis-hominis* UM 256 predicted by MEROPS. **Table L in S1 File**. List of *ERG11/CYP51* enzymes that involved in antifungal drug resistance in *P*. *unguis-hominis* UM 256. **Table M in S1 File**. List of MFS transport subfamilies, DHA 1 and DHA 2 predicted in *P*. *unguis-hominis* UM 256. **Table N in S1 File**. List of multidrug resistance genes of *P*. *unguis-hominis* UM 256. **Table O in S1 File**. List of predicted antifungal resistance genes in *P*. *unguis-hominis* UM 256 and other dematiaceous fungi.
(XLSX)
