# Supplementary material for: Genome Anatomy of Pyrenochaeta unguis-hominis UM 256, a Multidrug Resistant Strain Isolated from Skin Scraping
Source: PLoS One. 2016 Sep 14;11(9):e0162095. doi: 10.1371/journal.pone.0162095 (PMC5023194; doi:10.1371/journal.pone.0162095)
Supplement: S1 Fig — (A) ERG11/CYP51 genes. Lane 3, lane 5, lane 7 and lane 9 are the negative control. The PCR products for ERG 11/ CYP51 genes (lane 4: UM256_11225, lane 5: UM256_2977, lane 8: UM256_2978, and lane 10: UM256_1421) showed targeted single band. (B) CDR1 genes. Lane 2, lane 4, lane 6, lane 8 and lane 10 are the negative control. The PCR products for CDR1 genes (lane 1: UM256_9423, lane 5: UM256_5932, lane 7: UM256_11463, and lane 9: UM256_3801) showed targeted single band. However, PCR products at lane 6 (UM256_7510) showed no band. (C) CDR2 genes. Lane 1, and lane 3 are the negative control. The PCR products for CDR2 genes (lane 2: UM256_3553 and lane 4: UM256_5895) showed targeted single band. (D) MDR1 genes. Lane 1, and lane 3 are the negative control. The PCR products for MDR1 genes (lane 2: UM256_8687, and lane 4: UM256_11980) showed targeted single band. (E) MDR2 genes. Lane 2 and lane 4 are the negative control. The PCR products for MDR2 genes (lane 1: UM256_7395, and lane 3: UM256_8619) showed targeted single band. The ladder used for (A) and (E) is 500 bp DNA ladder (iDNA Biotechnology, Malaysia) and the ladder used for (B), (C) and (D) is 1kb DNA ladder (Thermo scientific, US). (PDF) [file pone.0162095.s001.pdf]

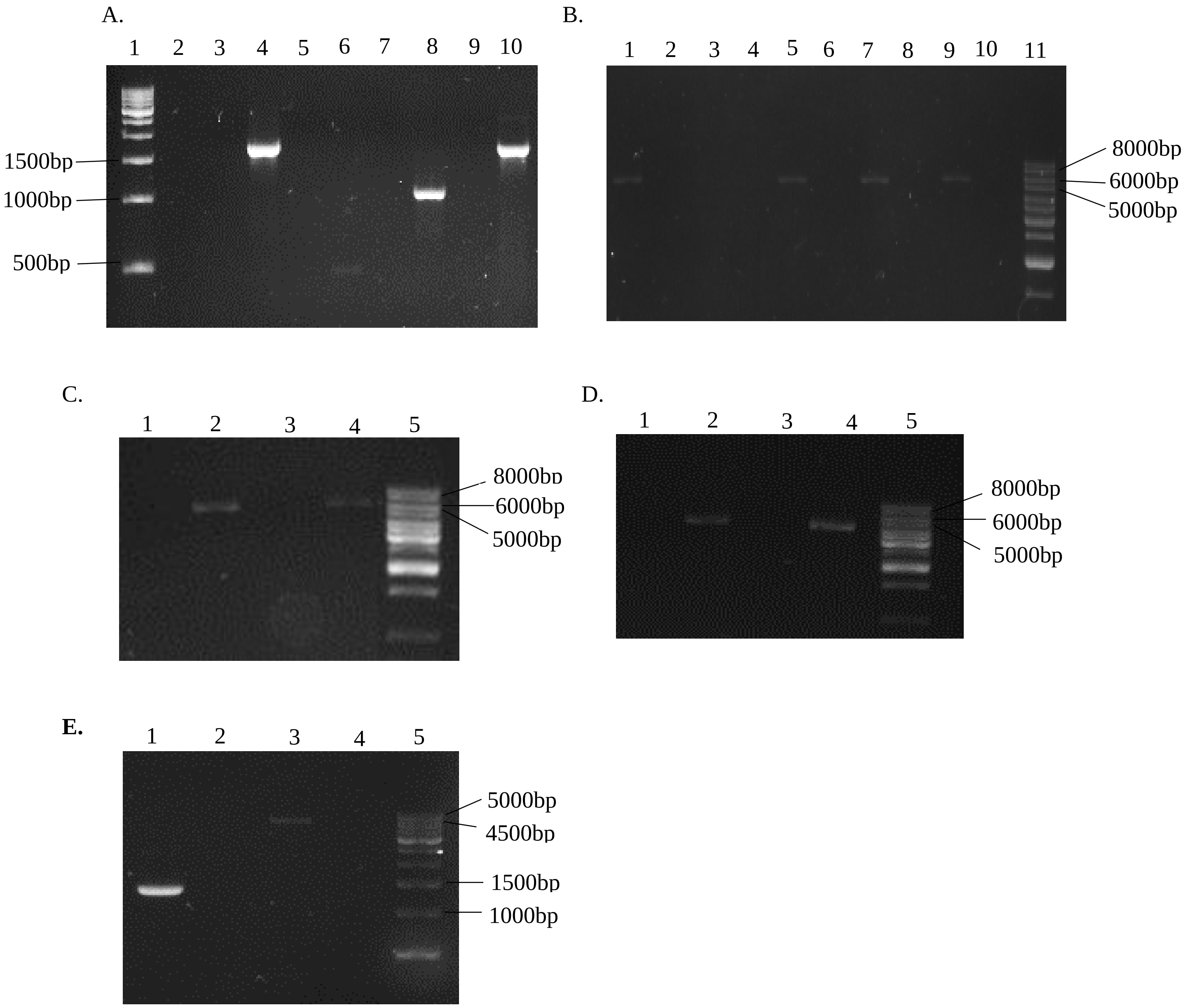


**S1 Fig: PCR amplification of azole resistance genes. (A) *ERG11/CYP51* genes.** Lane 3, lane 5, lane 7 and lane 9 are the negative control. The PCR products for *ERG 11/ CYP51* genes (lane 4: UM256_11225, lane 5: UM256_2977, lane 8: UM256_2978, and lane 10: UM256_1421) showed targeted single band. **(B) *CDR1* genes.** Lane 2, lane 4, lane 6, lane 8 and lane 10 are the negative control. The PCR products for *CDR1* genes (lane 1: UM256_9423, lane 5: UM256_5932, lane 7: UM256_11463, and lane 9: UM256_3801) showed targeted single band. However, PCR products at lane 3 (UM256_7510) showed no band. **(C) *CDR2* genes.** Lane 1, and lane 3 are the negative control. The PCR products for CDR2 genes (lane 2: UM256_3553 and lane 4: UM256_5895) showed targeted single band. **(D) *MDR1* genes.** Lane 1, and lane 3 are the negative control. The PCR products for *MDR1* genes (lane 2: UM256_8687, and lane 4: UM256_11980) showed targeted single band. **(E) *MDR2* genes**. Lane 2 and lane 4 are the negative control. The PCR products for *MDR2* genes (lane 1: UM256_7395, and lane 3: UM256_8619) showed targeted single band. The ladder used for (A) and (E) is 500 bp DNA ladder (iDNA Biotechnology, Malaysia) and the ladder used for (B), (C) and (D) is 1kb DNA ladder (Thermo scientific, US).
