# Supplementary material for: Genome Anatomy of Pyrenochaeta unguis-hominis UM 256, a Multidrug Resistant Strain Isolated from Skin Scraping
Source: PLoS One. 2016 Sep 14;11(9):e0162095. doi: 10.1371/journal.pone.0162095 (PMC5023194; doi:10.1371/journal.pone.0162095)
Supplement: S2 Fig — The point mutation site are shown (arrow) in ERG11 genes (A) UM256_11225 (B) UM256_2977 (C) UM256_2978. (PDF) [file pone.0162095.s002.pdf]

A. UM256_11225

```
C.albican_X13296      MAIVETVIDGINYFLSLSVTQ-----QISILLGVPFVYNLVWQYLYSLRKDRAPLVFYWI 55
C.albican_AF153846    MAIVETVIDGINYFLSLSVTQ-----QISILLGVPFVYNLVWQYLYSLRKDRAPLVFYWI 55
UM256_11225           MGVLAHVAGPLGDFTSNSSRPVVFAAGFASFVLLAVVLN-VLKQLLPRKSNEPPLVFHWL 59
                      *.::  *   :  * * *         :: :: : .* * * : *   :.:. ****:*:

C.albican_X13296      PWFGSAASYGQQPYEFFESCRQKYGDVFSFMLLGKIMTVYLGPKGHEFVFNAKLSDVSAE 115
C.albican_AF153846    PWFGSAASYGQQPYEFFESCRQKYGDVFSFMLLGKIMTVYLGPKGHEFVFNAKLSDVSAE 115
UM256_11225           PVIGNTVTYGMDPYAFFFANQKKYGNVFTFILLGRKMTVCLDTTGNNFILNGKIKDVNAE 119
                      * :*.:.:** :** ** : ::***:**:*:***: *** *  .*.:*::*.*:.**.**

C.albican_X13296      DAYKHLTTPVFGKGVIYDCPNSRLMEQKKFAKFALTTDSFKRYVPKIREEILNYFVTDES 175
C.albican_AF153846    EAYKHLTTPVFGTGVIYDCPNSRLMEQKKFAKFVLTTDSFKRYVPKIREEILNYFVTDES 175
UM256_11225           EIYAPLTTPVFGKDVVYDCPNSKLMEQKKFVKFGLTQDALRSYVTLITQEVEDFVKRHKS 179
                      : *  *******. *:******:*******.** ** *::: **  * :*: ::.  .:*

C.albican_X13296      FKLKEKTHGVANVMKTQPEITIFTASRSLFGDEMRRIFDRSFAQLYSDLDKGFTPINFVF 235
C.albican_AF153846    FKLKEKTHGVANVMKTQPEITIFTASRSLFGDEMRRIFDRSFAQLYSDLDKGFTPINFVF 235
UM256_11225           FKGQKGT---FDVTKVMAELTIYTASRSLQGQEIRNSFDSRFAELYHDLDMGFSPVNFML 236
                      ** :: *    :* *.  *:**:****** *:*:*. **  **:** *** **:*:**::

C.albican_X13296      PNLPLPHYWRRDAAQKKISATYMKEIKSRRERGDIDPNRDLIDSLLIHSTYKDGVKMTDQ 295
C.albican_AF153846    PNLPLPHYWRRDAAQKKISATYMKEIKSRRERGDIDPNRDLIDSLLIHSTYKDGVKMTDQ 295
UM256_11225           SWAPLPHNRARDNARETMIKLYSELVRKRRSGEARKDGEHDMIWHLMECKYKDGTEVPEH 296
                         ****   ** *::.:   * : ::.**.     .  .. :   *:...****.:: ::

C.albican_X13296      EIANLLIGILMGGQHTSASTSAWFLLHLGEKPHLQDVIYQEVVELLKEKGGDLNDLTYED 355
C.albican_AF153846    EIANLLIGILMGGQHTSASTSAWFLLHLGEKPHLQDVIYQEVVELLKEKGGDLNDLTYED 355
UM256_11225           EIAGIMIALLMAGQHSSSSTIAWILLRLAQNSHLIEELLAEQK---AVLGEDLPPLTYED 353
                      *** ::*.:**.***:*:** **:**:*.:: ** : :  *         * **  *****

C.albican_X13296      LQKLPSVNNTIKETLRMHMPLHSIFRKVTNPLRIPETNYIVPKGHYVLVSPGYAHTSERY 415
C.albican_AF153846    LQKLPSVNNTIKETLRMHMPLHSIFRKVTNPLRIPETNYIVPKGHYVLVSPGYAHTSERY 415
UM256_11225           LQRLPLHAQTVKETLRLHAPIHSIMRKVKQPLVVDGTNYVVPTSHTLMSSPGFSAQLDTH 413
                      **:**    :*:*****:* *:***:***.:** :  ***:**..* :: ***::    : :

C.albican_X13296      FDNPEDFDPTRWDTAAAKANSVSFNSSDEVDYGFGKVSKGVSSPYLPFGGGRHRCIGEQF 475
C.albican_AF153846    FDNPEDFDPTRWDTAAAKANSVSFNSSDEVDYGFGKVSKGVSSPYLPFGGGRHRCIGEQF 475
UM256_11225           FVNPAVWDPHRWDAGQQNYEEAEEKDEDKIDYGWGVVSKGTNSPYLPFGAGRHRCIGEQF 473
                      * **  :** ***:.   : :...  :..*::***:* ****..*******.**********

C.albican_X13296      AYVQLGTILTTFVYNLRWTI--DGYKVPDPDYSSMVVLPTEPAEIIWEKRETCMF      528
C.albican_AF153846    AYVQLGTILTTFVYNLRWTI--DGYKVPDPDYSSMVVLPTEPAEIIWEKRETCMF      528
UM256_11225           AYLQLQTILVAFVREFKLRNVGGSKDIVGTDYSSLFSRPLAPGVVEWERREKA--      526
                      **:** ***.:** :::       . .:   ****:.  *  *. : **:**..
```

B. UM256_2977

```
UM256_2977           ---MPSP---------LN-LASAFLVLCIAYVAANIARQLLSLNKNEPPVVFHWLPWLGS  47
C.albican_X13296     MAIVETVIDGINYFLSLSVTQQISILLGVPFVYNLVWQYLYSLRKDRAPLVFYWIPWFGS  60
C.albican_AF153846   MAIVETVIDGINYFLSLSVTQQISILLGVPFVYNLVWQYLYSLRKDRAPLVFYWIPWFGS  60
                        : :          *.   .  ::* : :*   : : * **.*:. *:**:*:**:**
                                                                  ↓           ↓
UM256_2977           AVSYGRDPYKFLFAARAKHGDVFTFVLLGRNVTVHLGVAGNDFVFNGKETHINAEDIYGP  107
C.albican_X13296     AASYGQQPYEFFESCRQKYGDVFSFMLLGKIMTVYLGPKGHEFVFNAKLSDVSAEDAYKH  120
C.albican_AF153846   AASYGQQPYEFFESCRQKYGDVFSFMLLGKIMTVYLGPKGHEFVFNAKLSDVSAEEAYKH  120
                     *.***::**:*: :.* *:****:*:***: :**:**  *.:****.* :.:.**: *

UM256_2977           LTTPVFGKDVVYDCPNAKLMEQKKFVKFGLTTEALKAHRG--------------------  147
C.albican_X13296     LTTPVFGKGVIYDCPNSRLMEQKKFAKFALTTDSFKRYVPKIREEILNYFVTDESFKLKE  180
C.albican_AF153846   LTTPVFGTGVIYDCPNSRLMEQKKFAKFVLTTDSFKRYVPKIREEILNYFVTDESFKLKE  180
                     *******. *:*****::*******.** ***:::* :

UM256_2977           ------------------------------------------------------------  147
C.albican_X13296     KTHGVANVMKTQPEITIFTASRSLFGDEMRRIFDRSFAQLYSDLDKGFTPINFVFPNLPL  240
C.albican_AF153846   KTHGVANVMKTQPEITIFTASRSLFGDEMRRIFDRSFAQLYSDLDKGFTPINFVFPNLPL  240


UM256_2977           ------------------------------------------------------------  147
C.albican_X13296     PHYWRRDAAQKKISATYMKEIKSRRERGDIDPNRDLIDSLLIHSTYKDGVKMTDQEIANL  300
C.albican_AF153846   PHYWRRDAAQKKISATYMKEIKSRRERGDIDPNRDLIDSLLIHSTYKDGVKMTDQEIANL  300


UM256_2977           ------------------------------------------------------------  147
C.albican_X13296     LIGILMGGQHTSASTSAWFLLHLGEKPHLQDVIYQEVVELLKEKGGDLNDLTYEDLQKLP  360
C.albican_AF153846  LIGILMGGQHTSASTSAWFLLHLGEKPHLQDVIYQEVVELLKEKGGDLNDLTYEDLQKLP   360


UM256_2977           ------------------------------------------------------------  147
C.albican_X13296     SVNNTIKETLRMHMPLHSIFRKVTNPLRIPETNYIVPKGHYVLVSPGYAHTSERYFDNPE  420
C.albican_AF153846   SVNNTIKETLRMHMPLHSIFRKVTNPLRIPETNYIVPKGHYVLVSPGYAHTSERYFDNPE  420


UM256_2977           ------------------------------------------------------------  147
C.albican_X13296     DFDPTRWDTAAAKANSVSFNSSDEVDYGFGKVSKGVSSPYLPFGGGRHRCIGEQFAYVQL  480
C.albican_AF153846   DFDPTRWDTAAAKANSVSFNSSDEVDYGFGKVSKGVSSPYLPFGGGRHRCIGEQFAYVQL  480


UM256_2977           ------------------------------------------------ 147
C.albican_X13296     GTILTTFVYNLRWTIDGYKVPDPDYSSMVVLPTEPAEIIWEKRETCMF 528
C.albican_AF153846   GTILTTFVYNLRWTIDGYKVPDPDYSSMVVLPTEPAEIIWEKRETCMF 528
```

C. UM256_2978

```
C.albican_X13296       MAIVETVIDGINYFLSLSVTQQISILLGVPFVYNLVWQYLYSLRKDRAPLVFYWIPWFGS 60
C.albican_AF153846     MAIVETVIDGINYFLSLSVTQQISILLGVPFVYNLVWQYLYSLRKDRAPLVFYWIPWFGS 60
UM256_2978             ------------------------------------------------------------ 0


C.albican_X13296       AASYGQQPYEFFESCRQKYGDVFSFMLLGKIMTVYLGPKGHEFVFNAKLSDVSAEDAYKH 120
C.albican_AF153846     AASYGQQPYEFFESCRQKYGDVFSFMLLGKIMTVYLGPKGHEFVFNAKLSDVSAEEAYKH 120
UM256_2978             ------------------------------------------------------------ 0


C.albican_X13296       LTTPVFGKGVIYDCPNSRLMEQKKFAKFALTTDSFKRYVPKIREEILNYFVTDESFKLKE 180
C.albican_AF153846     LTTPVFGTGVIYDCPNSRLMEQKKFAKFVLTTDSFKRYVPKIREEILNYFVTDESFKLKE 180
UM256_2978             ------------------------------------------------------------ 0

                                                                  ↓
C.albican_X13296       KTHGVANVMKTQPEITIFTASRSLFGDEMRRIFDRSFAQLYSDLDKGFTPINFVFPNLPL 240
C.albican_AF153846     KTHGVANVMKTQPEITIFTASRSLFGDEMRRIFDRSFAQLYSDLDKGFTPINFVFPNLPL 240
UM256_2978             -----------MAQITIFTAASALLGPEVRSKLNNGFASLYHDLDGGFSPINFVLPHAPF 49
                                  :******: :*:* *:*  ::..**.** *** **:*****:*. *:
                                 ↓
C.albican_X13296       PHYWRRDAAQKKISATYMKEIKSRRERGDIDPNRDLIDSLLIHSTYKDGVKMTDQEIANL 300
C.albican_AF153846     PHYWRRDAAQKKISATYMKEIKSRRERGDIDPNRDLIDSLLIHSTYKDGVKMTDQEIANL 300
UM256_2978             PQNIKRDRAQVTMRKIYEGIIAD-RRAGKAPPTTDMIS-HLMQCSYKDGRPVPDKEISNM 107
                       *:  :** ** .:   *   * . *. *.  *. *:*.  *::.:****  : *:**:*:

C.albican_X13296       LIGILMGGQHTSASTSAWFLLHLGEKPHLQDVIYQEVVELLKEKGGDLNDLTYEDLQKLP 360
C.albican_AF153846     LIGILMGGQHTSASTSAWFLLHLGEKPHLQDVIYQEVVELLKEKGGDLNDLTYEDLQKLP 360
UM256_2978             MITILMAGQHNSSNVASWIMLHLANEPQICEELYQEQLDQLADENGSLPELELRDMEKLQ 167
                       :* ***.***.*:..::*::***.::*:: : :*** :: * :: *.* :*  .*::**

C.albican_X13296       SVNNTIKETLRMHMPLHSIFRKVTNPLRIPETNYIVPKGHYVLVSPGYAHTSERYFDNPE 420
C.albican_AF153846     SVNNTIKETLRMHMPLHSIFRKVTNPLRIPETNYIVPKGHYVLVSPGYAHTSERYFDNPE 420
UM256_2978             LHSNVVKETLRMHNAIHSIMRLVKRPLPVPGTPWTIPPGHAVLASPGVSANSEEYFPNPT 227
                         .*.:*******  :***:* *..** :* * : :* ** **.*** : .**.** **
                                          ↓         ↓
C.albican_X13296       DFDPTRWDTAAAKANSVSFNSSDEVDYGFGKVSKGVSSPYLPFGGGRHRCIGEQFAYVQL 480
C.albican_AF153846     DFDPTRWDTAAAKANSVSFNSSDEVDYGFGKVSKGVSSPYLPFGGGRHRCIGEQFAYVQL 480
UM256_2978             KWDPHRWDNNRDIE---EDGESDMVDYGYGRTSRGTKSAYLPFGGGRHRCMGEKFAHLNL 284
                       .:** ***.         .   .** ****:*:.*:*..* ***********:**:**:::*

C.albican_X13296       GTILTTFVYNLRWT--IDGYKVPDPDYSSMVVLPTEPAEIIWEKRETCMF       528
C.albican_AF153846     GTILTTFVYNLRWT--IDGYKVPDPDYSSMVVLPTEPAEIIWEKRETCMF       528
UM256_2978             EVITAVMVRTFRFKNIDGREGVPGTDYSSMLSRPLEPAEICWERRRAEST       334
                        .* :.:* .:*:.        **  *****:  * ***** **:*.:
```

S2 Fig. Alignment sequences of *ERG11/CYP51* genes sequence of UM256 with two *C. albicans* (GenBank accession number X13296 and AF153846). The point mutation site are shown (arrow) in *ERG*11 genes (A) UM256_11225 (B) UM256_2977 (C) UM256_2978
