# Supplementary material for: Genome Anatomy of Pyrenochaeta unguis-hominis UM 256, a Multidrug Resistant Strain Isolated from Skin Scraping
Source: PLoS One. 2016 Sep 14;11(9):e0162095. doi: 10.1371/journal.pone.0162095 (PMC5023194; doi:10.1371/journal.pone.0162095)
Supplement: S3 Fig — Point mutations was shown in FKS “hot-spot” 1 regions of UM256_4224, C. albicans and A. fumigatus. (PDF) [file pone.0162095.s003.pdf]

```
C.albicans      1 GYYQQQ----------------YDDMGQQPHQQDYYDPNAQYQQQPYDMDGYQDQANYGG
UM 256          1 AYYDESAYYDGQQGGHYQQNGYYDDRGQQGYQDEYY--NDQYYDQGGAQDGYAQQPRRRN
A.fumigatus     1 GYYDRSGYY-GPDGNHNQQEGGYYDAGQP--HDDYY--GDHYYDQGNGQQGYDNRGRRRG

C.albicans     45 QPMNAQGYNADPEAFSDFSYGGQTP----------GTPGYDQYGT--------QYTP--S
UM256          59 -----HDSEEDSETFSDFTMRSDMARATDMDYYGRGDERYNSYGD----GNNRGFRPPSS
A.fumigatus    56 ------DSEEDSETFSDFTMRSETARAADMDYYGRGDERYNSYADSQYGGRGYGYRPPSS

C.albicans     85 QMSYGGDPRSSGASTPIYGGQGQGYDPTQFNMSSNLPYPAWSADPQAPIKIEHIEDIFID
UM256         110 QVSYGGN-RSSGASTPIYGMDYSNALPA--GQRSREPYPAWTAEAQIPCTKEEIEDIFLD
A.fumigatus   110 QISYGAN-RSSGASTPVYGMDYGNALPA--GQRSREPYPAWASDGQVPVSKEEIEDIFLD

C.albicans    145 LTNKFGFQRDSMRNMFDYFMTLLDSRSSRMSPAQALLSLHADYIGGDNANYRKWYFSSQQ
UM256         167 LTAKFGFQRDSMRNMYDHFMTLLDSRASRMSPNQALLSLHADYIGGENANYRRWYFAAHL
A.fumigatus   167 LVNKFGFQRDSMRNMYDHLMTMLDSRASRMTPNQALLSLHADYIGGDNANYRRWYFAAHL

C.albicans    205 DLDDSLGFANMTLGKIGRKARKASKKSKKARKAAEEHGQDVDALANELEGDYSLEAAEIR
UM256         227 DLDDAVGFANMNLGKANRRTRKA---RKAAKKKASENPGNEQETLDAYEGDNSLEAAEYR
A.fumigatus   227 DLDDAVGFANMKLGKADRKTRKA---RKAAKKAAQQNPENVEETLEALEGDNSLEAAEYR

C.albicans    265 WKAKMNSLTPEERVRDLALYLLIWGEANQVRFTPECLCYIYKSATDYLNSPLCQQRQEPV
UM256         284 WKTRMNRMSQNDRVRQIALYLLCWGEANQVRFMPEVLCFIFKCADDYLNSPAGQAQTEPI
A.fumigatus   284 WKTRMNKMSQHDRVRQLALFLLCWGEANQVRFLPECLCFIFKCADDYYNSPECQNRVEPV

C.albicans    325 PEGDYLNRVITPLYRFIRSQVYEIYDGRFVKREKDHNKVIGYDDVNQLFWYPEGISRIIF
UM256         344 EEFTYLNEVITPLYQYCRDQGYEIQDGKYVRRERDHAAIIGYDDINQLFWYPEGLERIVF
A.fumigatus   344 EEFTYLNEIITPLYQYCRDQGYEIVDGKYVRRERDHNQII-VSDMNQLFWYPEGIERIAL

C.albicans    385 EDGTRLVDIPQEERFLKLGEVEWKNVFFKTYKEIRTWLHFVTNFNRIWIIHGTIYWMYTA
UM256         404 EDKSRIVDLPPAERYSKLKDVLWKKVFFKTYYERRSWFHMLVNFNRIWVIHVTSFWFYTA
A.fumigatus   403 EDKTRLVDIPPAERWTKLKDVVWKKAFFKTYKETRSWFHMITNFNRIWVIHLGAFWFFTA

C.albicans    445 YNSPTLYTKHYVQTINQQPLASSRWAACAIGGVLASFIQILATLFEWIFVPREWAGAQHL
UM256         464 YNSQPIYTKNYQQQLDQRPDKAATLSAVALGGTIASLIQIFATLAEWAYVPRKWAGAQHL
A.fumigatus   463 FNAQSLYTDNYQQQVNNKPPGYRIWSAVGFGGALSSFIQIAATICEWMYVPRRWAGAQHL

C.albicans    505 SRRMLFLVLIFLLNLVPPVYTFQITKLVIYSKS-AYAVSIVGFFIAVATLVFFAVMPLGG
UM256         524 TKRLLFLIAVFAVNIAPSVYIFGLDKTTG---TIANVLGGVQFAIALLTFIFFSIMPLGG
A.fumigatus   523 TKRLMFLILVFVINLAPGVFVFAYSKSMGISKTIPLIVGIVHFFVALATFVFFSVMPLGG

C.albicans    564 LFTSYMNKRSRRYIASQTFTANYIKLKGLDMWMSYLLWFLVFLAKLVESYFFSTLSLRDP
UM256         581 LFGSYLTRNSRKYVASQTFTASYPRLKGNDMWMSYGLWVLVFAAKLAESYFFLTLSIKDP
A.fumigatus   583 LFGSYLKKHGRQYVASQTFTASFPRLHGNDMWMSYGLWVCVFGAKLAESYFFLTLSFKDP
Conserved region                                                   FLTLSLRDP

C.albicans    624 IRNLSTMTM-RCVGEVWYKDIVCRNQAKIVLGLMYLVDLLLFFLDTYMWYIICNCIFSIG
UM256         641 IRILSHMKKPVCLGDAIFGDILCKYQPRILLGLMYFMDLVLFFLDSYLWYIIANMLFSVS
A.fumigatus   643 IRILSPMQIHQCAGVKYIGNVLCHKQPQILLGLMFFMDLTLFFLDSYLWYIICNTVFSVA

C.albicans    683 RSFYLGISILTPWRNIFTRLPKRIYSKILATTEMEIKYKPKVLISQIWNAIVISMYREHL
UM256         701 RSFYLGVSIWTPWRNIFSRLPKRIYSKILATTDMEIKYKPKVLISQIWNAVVISMYREHL
A.fumigatus   703 RSFYLGVSIWSPWRNIFSRLPKRIYSKVLATTDMEIKYKPKVLISQVWNAIIISMYREHL

C.albicans    743 LAIDHVQKLLYHQVPSEIEGKRTLRAPTFFVSQDDNNFETEFFPRNSEAERRISFFAQSL
UM256         761 LAIDHVQKLLYHQVPSEQEGKRTLRAPTFFVSQEDHSFKTEFFPAQSEAERRISFFAQSL
A.fumigatus   763 LAIDHVQKLLYHQVPSEQEGKRTLRAPTFFVSQEDQSFKTEFFPPGSEAERRISFFAQSL

C.albicans    803 ATPMPEPLPVDNMPTFTVFTPHYSEKILLSLREIIREDDQFSRVTLLEYLKQLHPVEWDC
UM256         821 STPIPEPLPVDNMPTFTVLIPHYGEKILLSLREIIREDEPYSRVTLLEYLKQLHPHEWDC
A.fumigatus   823 STPMPEPLPVDNMPTFTVLIPHYSEKILLSLREIIREDEPYSRVTLLEYLKQLHPHEWDC
```

```
C.albicans    863 FVKDTKILAEETAAYENGDDSEKLSEDGLKSKIDDLPFYCIGFKSAAPEYTLRTRIWASL
UM256         881 FVKDTKILADETSQF-NGDD-EKGEKDTAKSKIDDLPFYCIGFKSAAPEYTLRTRIWASL
A.fumigatus   883 FVKDTKILADETSQF-NGEP-EKSEKDVAKSKIDDLPFYCIGFKSAAPEYTLRTRIWSSL

C.albicans    923 RSQTLYRTVSGFMNYARAIKLLYRVENPELVQYFGGDPEGLELALERMARRKFRFLVSMQ
UM256         939 RSQTLYRTISGFMNYSRAIKLLYRVENPEVVQMFGGNSDKLERELERMARRKYKICVSMQ
A.fumigatus   941 RSQTLYRTVSGFMNYSRAIKLLYRVENPEVVQMFGGNSEKLERELERMARRKFKIVVSMQ

C.albicans    983 RLSKFKDDEMENAEFLLRAYPDLQIAYLDEEPALNEDEEPRVYSALIDGHCEMLENGRRR
UM256         999 RYAKFTKEERENTEFLLRAYPDLQIAYLDEEPPLAEGEEPRIYSALIDGHSEIMDNGMRR
A.fumigatus  1001 RYAKFNKEERENTEFLLRAYPDLQIAYLDEEPPVNEGEEPRLYSALIDGHCELLENGMRK

C.albicans   1043 PKFRVQLSGNPILGDGKSDNQNHAVIFHRGEYIQLIDANQDNYLEECLKIRSVLAEFEEM
UM256        1059 PKFRIQLSGNPILGDGKSDNQNHSIIFYRGEYIQLIDANQDNYLEECLKIRSVLAEFEEM
A.fumigatus  1061 PKFRIQLSGNPILGDGKSDNQNHSIIFYRGEYIQVIDANQDNYLEECLKIRSVLAEFEEL

C.albicans   1103 NVEHVNPYAPNLKSEDNNTKKDPVAFLGAREYIFSENSGVLGDVAAGKEQTFGTLFARTL
UM256        1119 TTDNVSPYTPGIPNPNFN----PVAILGAREYIFSENIGILGDIAAGKEQTFGTMFARTL
A.fumigatus  1121 TTDNVSPYTPGIPSTNTN----PVAILGAREYIFSENIGVLGDVAAGKEQTFGTLFARTL

C.albicans   1163 AQIGGKLHYGHPDFLNATFMLTRGGVSKAQKGLHLNEDIYAGMNAMMRGGKIKHCEYYQC
UM256        1175 AQIGGKLHYGHPDFLNGIFMTTRGGVSKAQKGLHLNEDIYAGMNALLRGGRIKHCEYYQC
A.fumigatus  1177 AQIGGKLHYGHPDFLNGIFMTTRGGISKAQKGLHLNEDIYAGMNAMIRGGRIKHCEYYQC

C.albicans   1223 GKGRDLGFGSILNFTTKIGAGMGEQMLSREYFYLGTQLPLDRFLSFYYGHPGFHINNLFI
UM256        1235 GKGRDLGFGSVLNFTTKIGTGMGEQMLSREYYYMGTQLPLDRFLSFYYAHPGFHINNVFI
A.fumigatus  1237 GKGRDLGFGSILNFTTKIGTGMGEQMLSREYYYLGTQLPLDRFLSFYYAHPGFHINNMFI

C.albicans   1283 QLSLQVFILVLGNLNSLAHEAIMCSYNKDVPVTDVLYPFGCYNIAPAVDWIRRYTLSIFI
UM256        1295 MLSVQCFMFVILHLGALHHETILCHFNKDIPITDPQWPNGCANLVPVFDWVWRCIVSIFI
A.fumigatus  1297 MLSVQMFMIVLINLGALKHETITCRYNPDLPITDPLRPTYCANLTPIVDWVNRCIISIFI

C.albicans   1343 VFFISFIPLVVQELIERGVWKAFQRFVRHFISMSPFFEVFVAQIYSSSVFTDLTVGGARY
UM256        1355 VFFISFVPLVVQELTERGFWRAATRMAKHFSSGSPFFEVFVTQIYANSLQTNLSFGGARY
A.fumigatus  1357 VFFISFVPLAVQELTERGVWRMAMRLAKHFGSVSFMFEVFVCQIYANAVHQNLSFGGARY

C.albicans   1403 ISTGRGFATSRIPFSILYSRFADSSIYMGARLMLILLFGTVSHWQAPLLWFWASLSALMF
UM256        1415 IGTGRGFATARIPFGILYSRFAGPSIYLGARALMMLLFATITVWGPWLIYFWLSLLSLCL
A.fumigatus  1417 IGTGRGFATARIPFGVLYSRFAGPSIYAGARSLLMLLFATSTVWTAALIWFWVSLLALCI

C.albicans   1463 SPFIFNPHQFAWEDFFLDYRDFIRWLSRGNTKWHRNSWIGYVRLSRSRITGFKRKLTGDV
UM256        1475 APFLFNPHQFSWDDFFIDYREYLRWLSRGNTRSHSASWIGYCRLSRTRITGYKRKILGDP
A.fumigatus  1477 SPFLFNPHQFAWNDFFIDYRDYLRWLSRGNSRSHASSWIGFCRLSRTRITGYKRKLLGVP

C.albicans   1523 SEKAAGDASRAHRSNVLFADFLPTLIYTAGLYVAYTFINAQTGV---TSYPYEINGSTDP
UM256        1535 TAKLSGDVPRAAFTNIFMSEIMGPLVLVAITIIPYLFINAQTGVNDERDTENEDQGLGNP
A.fumigatus  1537 SEKGSGDVPRARLTNIFFSEIIAPLVLVAVTLVPYLYINSRTGV---RD---------NP

C.albicans   1580 QPVNSTLRLIICALAPVVIDMGCLGVCLAMACCAGPMLGLCCKKTGAVIAGVAHGVAVIV
UM256        1595 KASGALVRVAIIAFGPIAVNAGVLGGLFALACCAGPLLSMCCKKFGAVLAAIAHAIAVIM
A.fumigatus  1585 ETTDAILRLAIVAAGPIAINAGVAGVFFGMACCMGPIFSMCCKKFGAVLAAIAHAIAVIV

C.albicans   1640 HIIFFIVMWVTEGFNFARLMLGIATMIYVQRLLFKFLTLCFLTREFKNDKANTAFWTGKW
UM256        1655 LLIFFVVMMFLEGFSFPRALAGMIAVVAIQRFFFKLIISLALTREFKADTANIAWWTGKW
A.fumigatus  1645 LLAIFEVMFFLESWSWPRMLIGMIAAAAIQRFIYKLIIALALTREFKHDQSNIAWWTGKW

C.albicans   1700 YNTGMGWMAFTQPSREFVAKIIEMSEFAGDFVLAHIILFCQLPLLFIPLVDRWHSMMLFW
UM256        1715 YT--MGWHTISQPGREYLCKITELGMFAADFILGHVLLFFMLPILLIPYADKFHSVMLFW
A.fumigatus  1705 YN--MGWHSMSQPGREFLCKITELGYFSADFVLGHVLLFAMLPALCVPFIDKFHSVMLFW
```

```
C.albicans   1760 LKPSRLIRPPIYSLKQARLRKRMVRKYCVLYFAVLILFIVIIVAPAVASGQIAVDQFANI
UM256        1773 LRPSRQIRPPIYSLKQTKLRKRRVIRYAILYFFLLVIFLALIVGPIVAGSKFKFD-----
A.fumigatus  1763 LRPSRQIRPPIYSLKQSKLRKRRVIRFAILYFGMLILFLVLLIAPLVVRSMGLVK-----

C.albicans   1820 GGSGSIADGLFQPRNVSNNDTGNHRPKTYTWSYLS---TRFTGSTTPYSTNPFRV-----
UM256        1828 --LPTLPMEILQPTGFNNNDT----KTTTTGRCLQGTCPKWDGDEDASSDDAETTEDASR
A.fumigatus  1818 --TPNLPFNLLQPLDKDNNDT----MVTYTGNNIP---AGFEPVESASSVATATS-----

C.albicans        --------
UM256        1882 RFRRYMAY
A.fumigatus       --------
```

S3 Fig. Alignment of UM256_4224, *C. albicans* (GenBank D88815) and *A. fumigatus* (GenBank U79728). Point mutations was shown in FKS “hot-spot” 1 regions of UM256_4224, *C. albicans* and *A. fumigatus*.
